# Cell Fate Regulation Governed by a Repurposed Bacterial Histidine Kinase

**DOI:** 10.1371/journal.pbio.1001979

**Published:** 2014-10-28

**Authors:** W. Seth Childers, Qingping Xu, Thomas H. Mann, Irimpan I. Mathews, Jimmy A. Blair, Ashley M. Deacon, Lucy Shapiro

**Affiliations:** 1 Department of Developmental Biology, Stanford University School of Medicine, Stanford, California, United States of America; 2 Stanford Synchrotron Radiation Lightsource, SLAC National Accelerator Laboratory, Menlo Park, California, United States of America; 3 Joint Center for Structural Genomics, United States of America; Rutgers University-Robert Wood Johnson Medical School, United States of America

## Abstract

One of the simplest organisms to divide asymmetrically is the bacterium *Caulobacter crescentus*. The DivL pseudo-histidine kinase, positioned at one cell pole, regulates cell-fate by controlling the activation of the global transcription factor CtrA via an interaction with the response regulator (RR) DivK. DivL uniquely contains a tyrosine at the histidine phosphorylation site, and can achieve these regulatory functions *in vivo* without kinase activity. Determination of the DivL crystal structure and biochemical analysis of wild-type and site-specific DivL mutants revealed that the DivL PAS domains regulate binding specificity for DivK∼P over DivK, which is modulated by an allosteric intramolecular interaction between adjacent domains. We discovered that DivL's catalytic domains have been repurposed as a phosphospecific RR input sensor, thereby reversing the flow of information observed in conventional histidine kinase (HK)-RR systems and coupling a complex network of signaling proteins for cell-fate regulation.

## Abstract 2

The pathway that regulates asymmetric cell division in Caulobacter involves a signaling kinase whose catalytic output domain has been repurposed as an input sensor of the phosphorylation state of the response regulator – a reversal of the conventional direction of information flow; this allows wiring of simple linear signaling pathways into complex eukaryote-like networks.

## Author Summary

Across all kingdoms of life the generation of cell-type diversity is the consequence of asymmetry at the point of cell division. The bacterium *Caulobacter crescentus* divides asymmetrically to produce daughter cells that have distinct morphology and behavior. As in eukaryotes, an unequal distribution of signaling proteins in daughter *Caulobacter* cells triggers the differential read-out of identical genomes. A critical interaction between two protein molecules – a protein kinase (DivL) and a response regulator (DivK) – is known to occur exclusively in one daughter cell and to thereby regulate differentiation. However, mapping the observed signaling interconnections that drive asymmetric division has been difficult to reconcile with traditional models of bacterial signaling. Here we determine how DivL detects and processes this DivK signal. Although DivL has an architecture that is typical of histidine kinases, which normally act by regulating the phosphorylation state of the appropriate response regulator, DivL's essential functions do not require kinase activity and DivL does not add or remove phosphate from DivK. Instead we find that DivL has converted its output kinase domain into an input sensor domain that specifically detects phosphorylated DivK, and we identify key features of DivL that underlie this specificity. This novel reassignment of sensory functions reverses the conventional kinase-to-response-regulator signaling flow and logically couples linear signaling pathways into complex eukaryote-like networks to regulate cell development.

## Introduction

**Figure 1:**
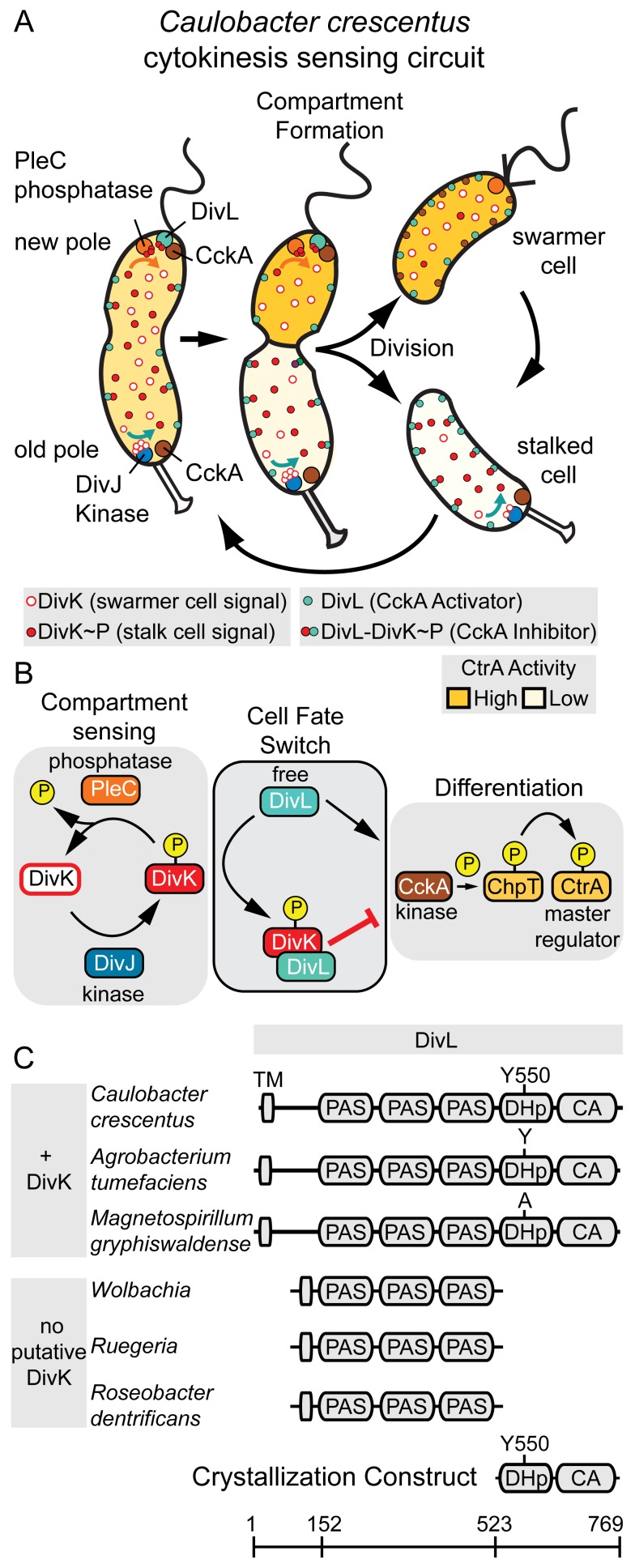
A compartment sensing circuit regulates asymmetric cell division in *Caulobacter crescentus*. (A) A cartoon of *C. crescentus* asymmetric cell division and the spatiotemporal localization of the essential regulatory components DivJ (blue), DivK (red), PleC (orange), CckA (brown), DivL (green), and CtrA (yellow). PleC serves as a DivK phosphatase localized at the new cell pole, while DivJ serves as a DivK kinase localized at the stalked cell pole. DivJ and PleC are physically separated upon compartmentalization, generating DivK∼P (full red circles) in the stalk compartment and DivK (open red circles) in the swarmer compartment. This compartment signal (DivK) selectively represses CtrA upon interaction with DivL thereby guiding distinct developmental programs for swarmer and stalked cells. (B) To promote cell differentiation, *Caulobacter* employs three distinct signaling modules: a compartment sensing module consisting of DivJ (HK), DivK (RR), and PleC (phosphatase), a differentiation module consisting of CckA (hybrid HK), ChpT (phosphotransferase), and CtrA (RR), and a cell-fate-switch module comprising the DivL pseudokinase that connects the compartment-sensing and differentiation modules. DivL activates the differentiation module when unbound (via CckA), but represses this module when bound to DivK∼P. (C) The domain organization of DivL in select α-proteobacteria that contain a putative DivK ortholog versus DivL domain organization in α-proteobacteria that do not contain a putative DivK ortholog [19]. Based on comparative analysis, DivL(523–769) containing only the DHp-CA domains was selected as a minimal DivK interacting unit for crystallization trials.

Cell fate decisions driven by the spatial organization of signaling proteins are a hallmark of asymmetric divisions in systems as diverse as stem cells and bacteria. *Caulobacter*, a model system for localization-dependent signaling network studies, divides asymmetrically yielding two distinct daughter cells: a motile swarmer cell and a replication-competent stalked cell (Figure 1). Cell division in *Caulobacter* is a stepwise process that includes a cytoplasmic compartmentalization stage [1] during which the inner membrane closes to form a barrier between the incipient swarmer and stalked cell compartments (Figure 1A) [2],[3]. This event segregates crucial signaling proteins [2],[4],[5] and culminates in the swarmer cell-specific activation of the global transcriptional regulator CtrA (Figure 1B) [6], which controls the expression of more than 100 genes involved in cell division and asymmetric polar organelle development [7]. The two daughter cells are distinguished by a compartment-sensing module composed of the response regulator (RR) DivK and two histidine kinases (HKs), DivJ and PleC, which differentially regulate the CtrA signaling pathway upon compartmentalization. DivJ and PleC respectively occupy the old and new cell poles (Figure 1A) [4],[5]. Prior to cell division, the cytoplasm contains a homogeneous mixture of phosphorylated (DivK∼P) and unphosphorylated DivK [8]. In pre-divisional cells, however, PleC (positioned at the new pole) functions as a DivK phosphatase, while DivJ (positioned at the old pole) functions as a DivK kinase [4],[5],[9]. In this way, compartmentalization promotes accumulation of unphosphorylated DivK in the incipient swarmer cell and DivK∼P in the incipient stalked cell [10],[11], providing unique cell-type markers.

In *Caulobacter*, the DivK phosphorylation state provides the basis for a regulatory switch that underlies divergent cell-fates upon cell division [12]–[15]. This switch is achieved through the activity of the pseudokinase DivL, which activates the CckA-ChpT-CtrA phosphorelay pathway in the uncomplexed state and inhibits this phosphorelay when bound to DivK∼P (Figure 1B) [13]. Previous *in vitro* work has shown that DivL has a binding preference for DivK∼P over unphosphorylated DivK, and genetic evidence suggests that the DivL-DivK∼P interaction leads to inhibition of the hybrid-HK CckA activity [13]. Additionally, DivL appears to play a role in the regulation of CckA polar localization and autokinase activity [13],[16]. Thus, DivL functions as a critical link that couples two distinct signaling pathways (Figure 1B).

 DivL has a predicted domain architecture that is typical of many HKs, consisting of multiple PAS domains fused to a C-terminal HK module that contains dimerization and histidine phosphotransfer (DHp) and catalytic and ATP-binding (CA) domains (Figure 1C). In contrast to typical HKs, a conserved tyrosine (Tyr550) appears in place of the predicted phosphorylatable histidine throughout DivL homologs in α-proteobacteria [17], with the exception of the *Magnetospirillum magneticum* DivL that contains a non-phosphorylatable alanine (Figure 1C). Furthermore, DivL's essential functions *in vivo* do not require a phosphorylated DHp domain [12],[13],[16], and the CA domain is dispensable for activity [12],[18]. Divergent α-proteobacteria that lack a DivK ortholog also frequently lack the catalytic domains (DHp-CA) of their DivL orthologs (Figure 1C) [19]. The co-evolution of DivK with the DHp-CA domains of DivL suggests that DivL and DivK may work together as a regulatory module within the cell-cycle [19]. Despite the apparent connection between DivK and DivL in cell fate signaling, the molecular nature of the interaction between this unconventional HK-RR pair is poorly understood. To gain insight into this signaling system, we solved the crystal structure of the HK region of DivL at 2.5 Å resolution and interrogated the interaction between DivL and both DivK and DivK∼P. Our results show that *Caulobacter* has repurposed a conserved HK as a highly specific sensor module for a phosphorylated RR∼P.

## Results

### DivL Binds Specifically to DivK∼P within the Core Cell-Cycle Signaling Circuit

**Figure 2:**
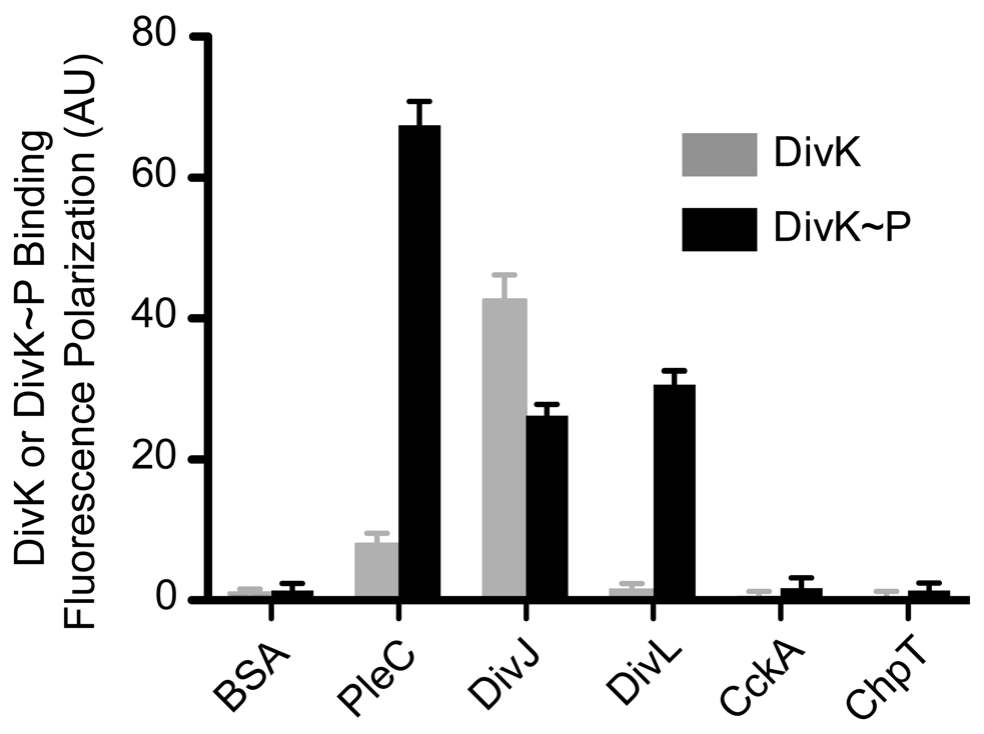
DivK binds specifically to PleC, DivJ, and DivL. Fluorescence polarization binding assay of BODIPY dye labeled DivK under unphosphorylated (gray) versus phosphorylated (black) conditions mixed with the following HKs at 10 µM PleC, DivJ, DivL, CckA, and ChpT. PleC and DivL binds to phosphorylated DivK specifically, while DivJ binds to both phosphorylated and unphosphorylated DivK. Numerical data used to generate manuscript graphs or histograms can be found in Table S1.

Previous experiments [13],[14], and our results obtained from *in vivo* co-immunoprecipitation analysis (Figure S1) all support an interaction between DivL and DivK. We employed a fluorescence polarization assay [20] to measure the binding affinities between DivL and DivK, as well as between DivK and other HKs in the compartment sensing signaling modules (Figure 1B, PleC and DivJ) and the differentiation module (Figures 1B, CckA and the ChpT histidine phosphotransfer protein, and 2). We fluorescently labeled DivK with a BODIPY dye and measured binding by mixing 10 µM of HK with 250 nM of labeled DivK or DivK∼P (see Materials and Methods). As shown in Figures 2 and S1, DivJ bound with similar affinity to both phosphorylated (K_d_ = 16±3.2 µM) and unphosphorylated (K_d_ = 8±2.1 µM) forms of DivK, whereas PleC preferentially bound to DivK∼P (K_d_ = 2.4±0.9 µM) over DivK (K_d_ = 26±30 µM). DivL(152–769) bound exclusively to DivK∼P with an apparent K_d_ of 11±4.2 µM, and very limited binding to unphosphorylated DivK (Figure S1). No binding of DivK to CckA or ChpT was observed regardless of the DivK phosphorylation state, even when the RR was present at concentrations as high as 100 µM. Thus, DivK or DivK∼P binds specifically to DivJ, PleC, and DivL with affinities within an order of magnitude of values reported for canonical HK-RR pairs (∼1 µM), while non-cognate pairs have binding affinities greater than ∼75 µM [21]. Thus, DivK impacts the CckA-ChpT-CtrA pathway without directly interacting with this set of signaling proteins.

### Structure of DivL's HK region

**Figure 3:**
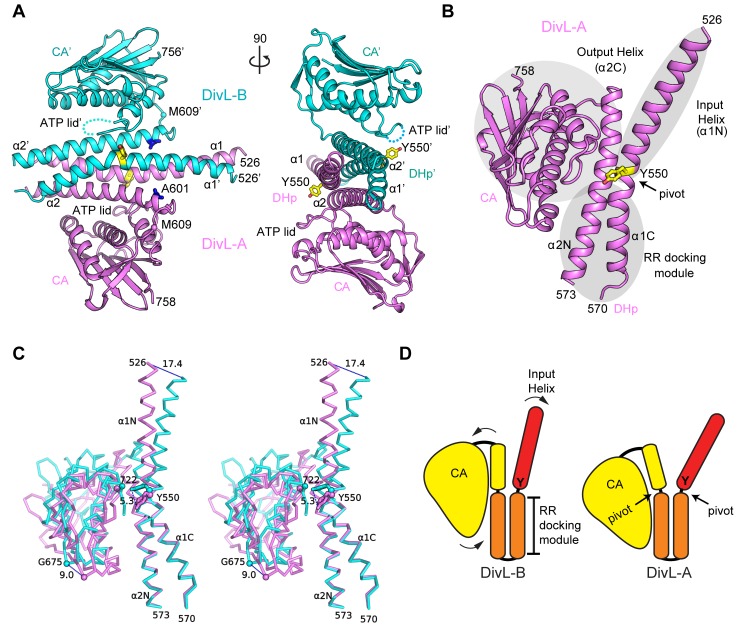
The DivL structure reveals an asymmetric dimer with conformational difference of monomers described as substructure rigid-body movements. (A) The DivL dimer. The monomeric subunits DivL-A and DivL-B are colored in violet and cyan, respectively. Tyr550 (yellow) and Ala601 (blue) are shown as sticks. (B) A monomer of DivL (here, DivL-A) consists of three rigid-body substructures (the “output helix+CA” module, input helix, and RR docking module, shaded in gray). (C) A stereoview of two DivL monomers superimposed about the RR docking module. Interatomic distances (in Å) are represented with dashed lines. (D) A schematic diagram showing the structural differences in two monomers (DivL-A and DivL-B) with substructures colored red (input helix), orange (RR docking module), and yellow (output helix+CA domain).

To define the structural basis for the phosphorylation-dependent binding of DivK to DivL, we determined the crystal structure of DivL's HK region (residues 523–769, Figures 1C and 3) by the multiple-wavelength anomalous diffraction (MAD) method using a gold derivative. The final model (PDB code 4q20) was refined to an R_cryst_ of 20.2% and an R_free_ of 23.2% using native data up to 2.5 Å resolution (Table S2). The model of DivL displays good geometry with an overall quality score that ranks in the 100th percentile compared to other structures with similar resolution, as calculated by MolProbity [22]. The asymmetric unit contains a DivL dimer (Figure 3A), which is consistent with size exclusion chromatography showing dimeric DivL in solution (observed MW = 50±15 kDa, predicted dimer MW = 47.3 kDa).

Overall, the DivL structure resembles other characterized HKs, such as the *Thermotoga maritima* HKs: HK853 [23],[24] and ThkA [25], although the DivL dimer is asymmetric. Asymmetric dimers were also observed in several recent HK structures (additional discussion included in Text S1) [26]–[29]. The dimer interface buries ∼3,900 Å^2^ total surface area. Each DivL monomer (DivL-A and DivL-B) consists of a DHp domain with two helices, α1 and α2 (residue range 526–608; since the α1–α2 loop is disordered in the crystal structure we assigned the connectivity of the two DHp helices in each monomer as in other class I HKs [23]), and a C-terminal CA domain with a highly conserved fold (residue range 609–758). The functionally important residue Tyr550 (colored in yellow in Figure 3A and 3B) is located at the middle of the first DHp helix (α1). Except for the substitution of a normally phosphorylatable histidine with tyrosine, DivL appears to retain all of the characteristic HK motifs, such as H, N, G1, F, and G2 boxes (Figure S2). A highly conserved patch of residues located near Tyr550 was revealed by mapping the degree of sequence conservation of DivL orthologs onto the DivL structure (Figure S2). We note with interest that this surface is typically involved in the RR recognition [23],[30].

### DivL Forms an Asymmetric Dimer

The CA domains of the DivL dimer are almost identical (RMSD of 0.64 Å for 144 C^α^ atoms). However, the DHp domains display significant structural differences (RMSD of 3.4 Å for 80 Cα atoms), indicating that the DivL dimer is asymmetric. The conformational differences between two DivL monomers are best described as rigid-body movements of substructures (Figure 3B–3D). Three rigid substructures can be identified in each monomer (Figure 3B), with each substructure representing a functional module. The largest substructure (residue range 591–758) consists of the CA domain and the “output helix,” which contains the C-terminal portion of α2 (α2C). The interface between α2C and CA is stabilized by clusters of highly conserved hydrophobic residues (Figure S3). A highly conserved CA domain and a very similar arrangement between α2C and CA can be identified in DivL, HK853, ThkA, and QseC (Figure S3), suggesting that this structural arrangement is a conserved HK feature. The second substructure (“RR docking module”) consists of the C-terminal portion of α1 (α1C) and the N-terminal portion of α2 (α2N) (Figure 3B); the equivalent region in HK853 is involved in its cognate RR recognition. [23]. The third substructure (“input helix”) consists of the first portion of α1 (α1N), which is connected to the N-terminal PAS sensor domains.

When two DivL monomers are superimposed about the RR docking module, the relative movements between the other two substructures become apparent (Figure 3C and 3D). The input helix in DivL-B is kinked more significantly around Tyr550 compared to DivL-A, resulting in a conformational change with respect to the Tyr550 side chain. This bending of the DHp helices is a common feature in many HKs [23],[26],[31],[32], due to the conserved residue Pro555 [32]. The “output helix+CA” module interacts and moves in tandem with the input helix, with a pivot point at the ATP binding site (proximal to Leu722). As a result, the bending of the input helix repositions the output+CA module with respect to the RR docking module, which may reflect HK conformational changes crucial for propagating long-range signals from the sensor domain to the catalytic domain or vise visa (Movies S1 and S2 illustrates the “morphing” between the two DivL conformations) and illustrated as a cartoon in Figure 3D. The structural movements produce an additional contact interface between DHp and CA in DivL-A, resulting in an increase in the buried surface area (1,730 Å^2^ total) compared to that of DivL-B (1,400 Å^2^ total) (Figure S3). The asymmetry of the DivL dimer likely reflects some inherent conformational flexibility of DivL in solution, even though it could be induced by the crystal packing. The temperature factor (i.e., B-values) distribution supports the assertion that the DivL structure is highly flexible, save for the central four-helix bundle near Tyr550 where there is less conformational freedom (Figure S3).

### DivL Interacts with DivK∼P Via the RR Docking Interface

**Figure 4:**
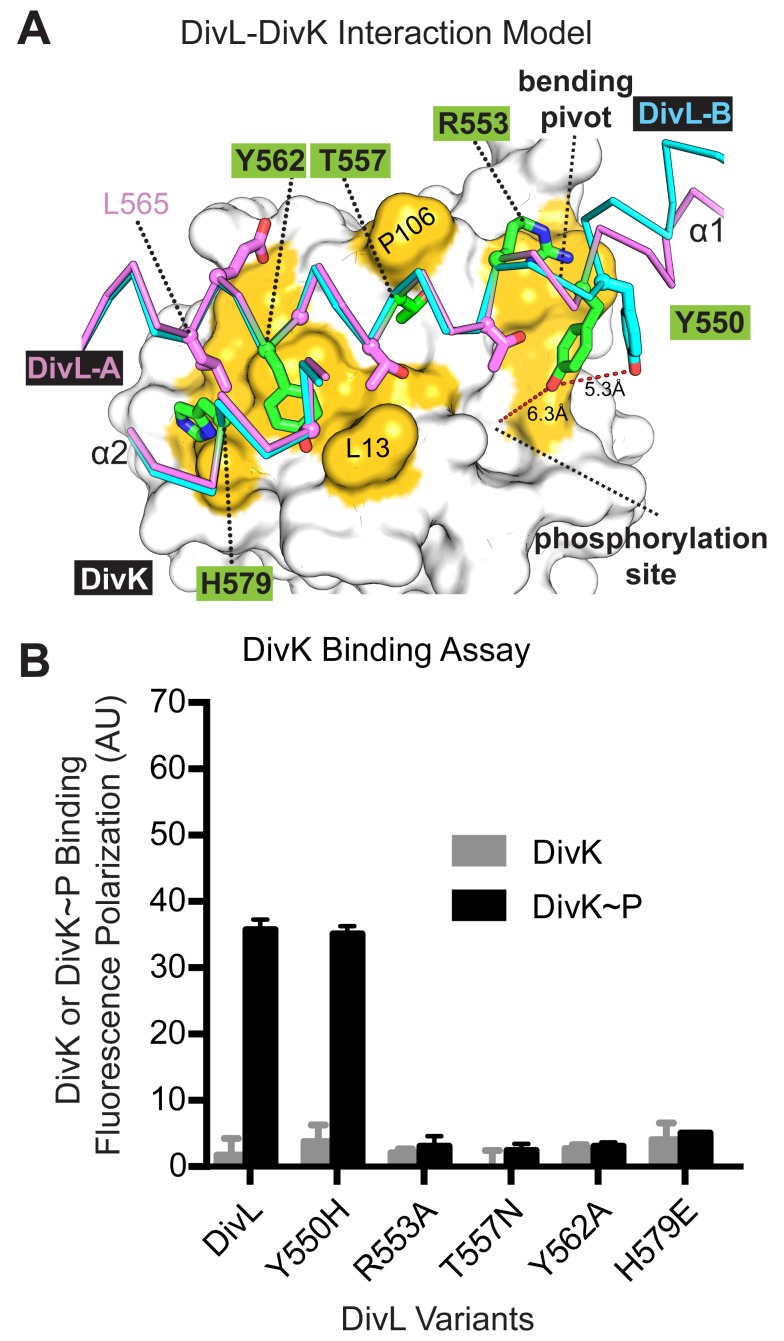
Point mutations in DivL's canonical HK-RR binding interface disrupt DivL-DivK∼P binding *in vitro*. (A) A computational model of the DivL-DivK∼P interface based on the HK853 complex with its cognate RD RR468 (PDB code 3dge). In this close-up view of the DivL-DivK∼P interaction DivK (PDB code 1mav) is shown as a white surface in the background with residues contacting DivL highlighted in yellow. The two forms of DivL are illustrated as violet (DivL-A) and cyan (DivL-B) Cα traces. Residues selected for site-directed mutagenesis are highlighted in green on DivL-A. (B) Fluorescence polarization binding assay examining the impact of point mutations (Y550H, R553A, T557N, Y562A, and H579E) on DivL-DivK and DivL-DivK∼P binding interaction. DivL variants R553A, T557N, Y562A, and H579E disrupt the binding to DivK, while DivL variant Y550H has no impact on DivK∼P binding. Numerical data used to generate manuscript graphs or histograms can be found in Table S1.

To build a computational model of the DivL-DivK∼P interaction, we compared the primary sequence of these proteins to the *T. maritima* HK-RR co-crystal structure of HK853-RR468 [23]. DivL's HK region shares 27% sequence identity with HK853, while DivK shares 36% sequence identity with RR468, the cognate RR for HK853. Strikingly, the surface of HK853 that directly interacts with RR468 is highly conserved in DivL, suggesting that the interaction between DivL and DivK is comparable to that observed between HK853 and RR468. Hence, we modeled a DivL-DivK∼P complex based on the HK853-RR468 co-complex (Figures 4A and S4). The resulting model places complementary surfaces of DivL and DivK∼P together with favorable interactions, supporting the model's feasibility. In this model, Leu565 and Gly561of DivL interact with a hydrophobic patch on the DivK∼P surface formed by Leu17, Leu21, Pro106, and Ile107. Furthermore, Ala582 of DivL makes an additional hydrophobic contact with Leu13 of DivK∼P. Potential hydrogen bonds between DivL and DivK are also predicted, such as Tyr562OH with Leu13O and Leu17N, Arg553N1 and Ala84O, and His579Nε2 with Asp20Oδ2.

To interrogate the predicted interface, we introduced targeted mutations (R553A, T557N, Y562A, H579E, and Y550H) within DivL at the putative DivK∼P docking site (Figure 4A, highlighted in green) and measured the effect of each mutation on the DivL-DivK∼P interaction. Each mutant was selected based upon introducing a significant side-chain perturbation, with the exception of T557N, which was selected based upon the presence of Asn (N) at this position in the DivJ kinase sequence. Each of these mutant proteins was purified and subjected to gel-filtration, which indicated that each variant formed dimers in solution. Four mutations (R553A, T557N, Y562A, and H579E) resulted in significant loss of DivK∼P binding (Figure 4B), with apparent dissociation constants measured to be 8–15-fold less than observed for wild-type DivL (Figure S5), highlighting the importance of these residues for binding of DivK∼P to DivL and supporting the validity of the DivL-DivK∼P computational model. The Y550H substitution did not reduce the affinity of DivL for DivK∼P. Intriguingly, T557N did not improve DivL's binding affinity for unphosphorylated DivK and instead diminished affinity for both forms of DivK. This indicates that DivL may bind DivK in a slightly different manner than DivJ. We also did not discover any mutations within this binding surface that switched binding specificity from DivL-DivK∼P to DivL-DivK.

Due to the asymmetric nature of the DivL dimer, Tyr550 of the DivL-A subunit (green in Figure 4A) is situated above the canonical phosphotransfer site (where the hydroxyl group may form hydrogen bond[s] with the phosphoryl group on Asp53 of DivK∼P), while in DivL-B (cyan in Figure 4A), both Tyr550 and the CA domain are displaced from the DivK active site (see Figure S4). This observation implies that conformational changes in DivL may impact affinity for DivK∼P. As a result, the asymmetric DivL dimer presents two different RR binding sites that share a common core surface but vary in the surface aspect involving Tyr550 and the ATP lid.

### PAS Sensor Domains Enhance Binding Specificity towards DivK∼P

**Figure 5:**
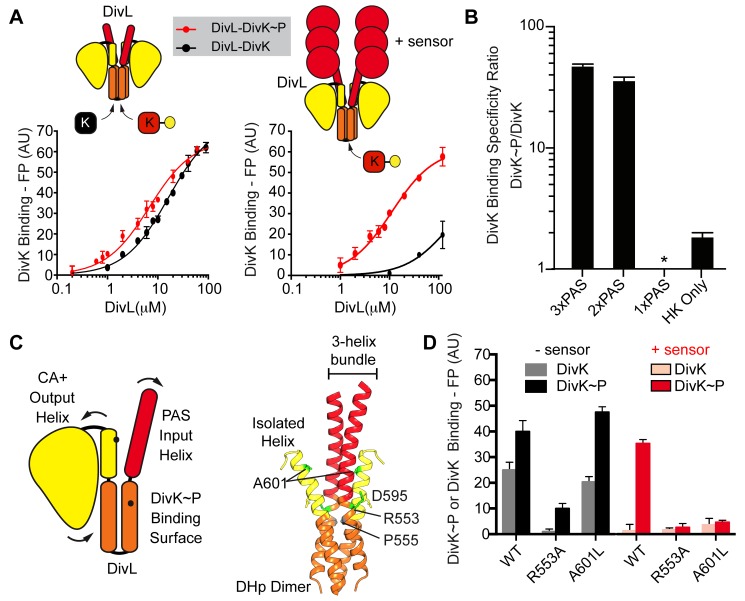
DivL sensor domains are critical for phosphospecific DivK recognition. (A) DivK (black) versus DivK∼P (red) binding curves for the DivL DHp-CA construct (no PAS domains) versus the DivL PAS 3X-DHp-CA construct (three PAS domains). (B) Relative amounts of DivK∼P versus DivK binding to DivL constructs containing 3, 2, 1, or 0 PAS domains. The minimal DivL construct that maintains phosphospecific DivK recognition contains two PAS domains. No DivL-DivK or DivL-DivK∼P binding was detected for the 1-PAS domain DivL construct. (C) Position of the allosteric point mutation A601L at the input-output helix interface. (D) Fluorescence polarization binding assay examining the impact of DivL point mutations in the DivL DHp-CA construct (black, left) versus the DivL PAS 3X-DHp-CA construct (red, right) upon both DivK and DivK∼P binding. The DivL(A601L) variant binds DivK∼P, but only when the PAS domains are deleted. Numerical data used to generate manuscript graphs or histograms can be found in Table S1.

DivL exhibits extensive structural homology with the PAS-linked HK ThkA [25], and comparative analysis suggests that a conserved surface-exposed patch on the DivL CA domain (Figure S2) could participate in a similar PAS domain interaction as observed in the ThkA structure. Because a PAS-CA interaction potentially restricts CA domain movement, we analyzed binding of DivK to DivL in the presence and absence of one or more DivL PAS domains to determine whether they influence the interaction between DivK and DivL. For these studies, we constructed DivL mutants containing 3, 2, 1, or 0 PAS domains and measured DivL-DivK or DivL-DivK∼P binding for each construct as a function of DivL concentration (0–120 µM). To our surprise, removal of all DivL PAS domains almost completely eliminated specificity for DivK∼P: DivL containing three PAS domains (residues 152–769) was highly selective for DivK∼P (K_d_ = 11 µM for DivK∼P and >150 µM for DivK), whereas a DivL variant with no PAS domains (i.e., crystallization construct) bound phosphorylated and unphosphorylated DivK with similar affinity (K_d_ = 15 µM for DivK and 7 µM for DivK∼P) (Figure 5A). DivL constructs containing three, and two PAS domains bind specifically to DivK∼P, while the 1-PAS domain DivL construct did not bind DivK∼P and may be less stable than other DivL constructs (Figure 5B). These results indicate that DivL's PAS sensor domains play a critical role in DivK∼P selectivity.

Previously, a DivL mutant (A601L) was shown to disrupt the DivL-DivK∼P interaction and thereby leading to the upregulation of CckA kinase activity *in vivo* [13]. Residue Ala601 lies at the interface between the input and output helices and away from the DivK∼P binding site (Figure 5C), indicating that the A601L mutation may impact DivL-DivK∼P binding through an allosteric effect rather than by modifying the docking site. Critically, this interface includes the C-terminal end of the DHp domain that has been shown to alter its structure by helical unwinding or bending to activate sensor kinases upon receiving a signal in the *Bacillus subtillus* HK KinA [33]. To determine whether the disruption of DivK∼P binding is due to perturbation of the RR module itself or of the arrangement of the PAS and CA domains with respect to the RR docking module, we evaluated the impact of A601L and a bona fide RR docking surface mutation (R553A) on DivK∼P binding to DivL that either contains or lacks PAS domains. We found that the R553A substitution disrupts DivK∼P binding regardless of whether the PAS domains are present. The DivL(A601L) variant binds DivK∼P and weakly to unphosphorylated DivK, but only when the PAS domains are deleted (Figure 5D). The observed PAS-domain-sensitive DivK∼P binding to DivL(A601L) suggests that interdomain interactions influence the binding of DivK∼P to DivL and explains in part the requirement for the PAS domains in DivK∼P selectivity. Specifically, in the DivL-A conformation the output helix forms a three-helix bundle in association with the two input helices (Figure 5C), whereas the output helix is isolated from the input domain in the DivL-B conformation (Figure 5C). Therefore, modulations of interactions at the input-output helix interface likely alters the PAS and CA inter-domain arrangement with respect to the DivK∼P binding site.

### DivL Lacks Kinase and Phosphatase Activities

Consistent with *in vivo* studies that indicate DivL's kinase activity is not essential for viability [12],[13],[16], we observed *in vitro* that DivL does not exhibit autokinase activity and that a Y550H mutation could not rescue activity (Figure S6). Loss of autokinase activity may be rooted in poor ATP binding as we found that DivL binds weakly to the ATP analog TNP-ATP with an apparent binding constant of 57±8 µM (Figure S7) [34]. DivL binds to TNP-ATP greater than 19× weaker than most prototypical HKs that exhibit binding constants in the range of 0.5–3 µM [34]–[37], with the exception of one study that reported a 294 µM Kd for the catalytic domains of PhoQ [38]. We also crystallized DivL in presence of various nucleotide analogs, but no nucleotide or magnesium ion could be identified in the density map (Figure S7). Structural analysis of the crystallized DivL conformation shows that both nucleotide-binding sites are either inaccessible or too small to accommodate ATP (Figure S7). Compared to other HKs, the DivL ATP lid mobility is more restricted since it is sandwiched between Tyr550 and the remainder of the CA domain. These results suggest that DivL has little intrinsic kinase activity compared to functional HK controls, which is consistent with our structural and nucleotide binding data.

**Figure 6:**
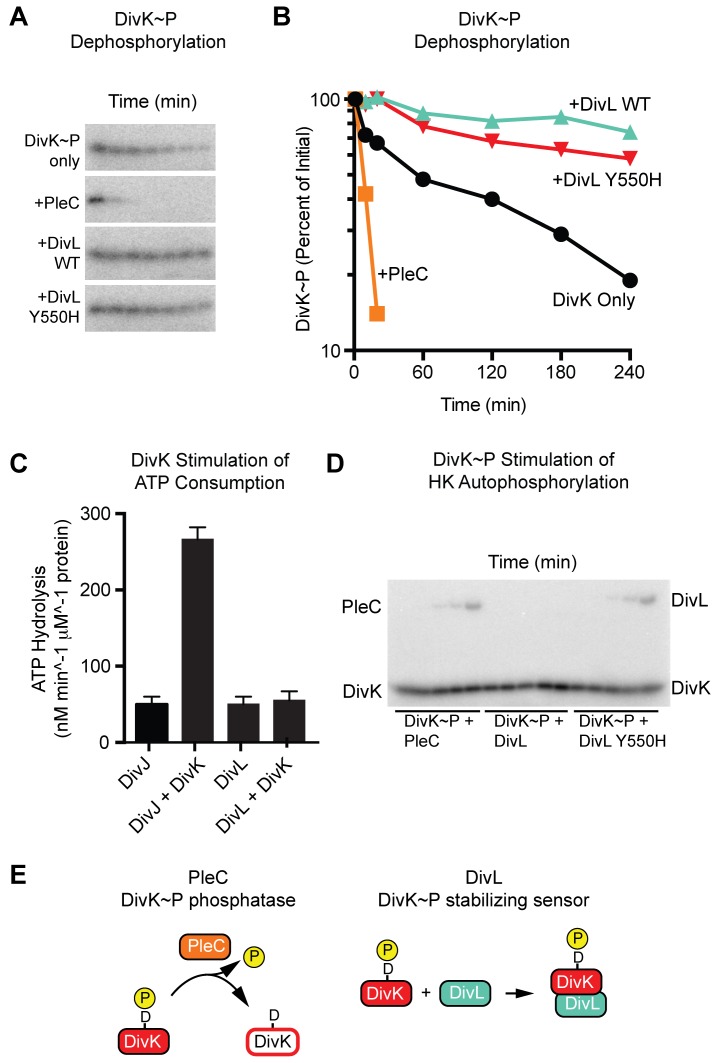
Functional interrogation of the DivL-DivK∼P interaction. (A) DivL-mediated stabilization of DivK∼P versus PleC-mediated dephosphorylation of DivK∼P is shown through a dephosphorylation time course (lane assignments: 1, 10, 20, 60, 120, 180, 240 minutes) with purified DivK∼P (1 µM) incubated alone, or together with 2.5 µM PleC or 2.5 µM DivL. Samples were transferred into SDS sample buffer and subjected to electrophoresis followed by phosphorimaging. (B) Quantification of DivK∼P decay from (A) in reactions containing DivK∼P alone (black) or DivK∼P with PleC (orange), or DivL (green), or DivL Y550H (red). (C) DivK stimulates DivJ ATP consumption, but has no impact on DivL ATP consumption. ATP consumption rates for DivJ, DivJ-DivK, DivL alone, and DivL-DivK are shown. (D) DivK∼P stimulates DivL Y550H autophosphorylation but has no impact on the phosphorylation state of wild-type DivL. Purified DivK∼P and [γ-^32^P]ATP were incubated with 5 µM PleC, with 5 µM DivL, or with 5 µM DivL Y550H for 0, 0.5, 1, 2, 5, and 20 minutes. (E) Cartoon illustrating the functional differences between the PleC phosphatase and the DivL phosphospecific DivK∼P sensor. Samples were transferred into SDS sample buffer and subjected to electrophoresis followed by phosphorimaging. Numerical data used to generate manuscript graphs or histograms can be found in Table S1.

 We also asked if DivL functions as a DivK phosphatase in a manner similar to PleC (Figure 6A and 6B). DivK∼P was generated by addition of His-DivJ, which was then purified by metal affinity chromatography. The remaining ATP in the DivK∼P reaction mixture was converted to ADP by incubation with hexokinase and glucose. DivK∼P phopho-aspartate stability was then compared over a 4 hour time frame in the presence of either PleC or DivL. We found that PleC reduced the DivK∼P half-life from 126 min to 8 min. However, the DivK∼P stability was increased in the presence of DivL (half-life >700 min). Furthermore, restoration of a phosphorylatable histidine on DivL (DivL Y550H) has no impact on DivK∼P phosphostability. Therefore, we conclude that DivL does not function as a DivK∼P phosphatase.

 It has been established that DivK allosterically activates the kinase activity of DivJ and PleC [9]. In light of these findings, we questioned whether any autokinase activity of DivL could be stimulated by DivK using a coupled-enzyme assay which assays ATPase activity (see Materials and Methods). Consistent with previous reports, we observed stimulation of DivJ autokinase activity in the presence of DivK. In contrast, no DivL autophosphorylation was observed upon addition of DivK or DivK∼P (Figure 6C). However, we did observe phosphate accumulation on DivL Y550H in the presence of DivK∼P and ATP (Figure 6D). In the absence of ATP no significant accumulation of phosphate signal was observed upon DivL Y550H (Figure S8), indicating that DivK∼P likely stimulate DivL autokinase activity in the presence of a canonical phosphate acceptor (i.e., His550). We do note it is formally possible DivK∼P has the ability to back transfer to DivL Y550H, but only in an ATP-dependent manner. Taken together, our results suggest that DivL does not act on DivK by modifying the RR phosphorylation state. Instead, based on previous genetic studies [13],[16], it appears the consequence of the DivL-DivK∼P interaction is to allosterically modulate DivL's regulatory functions towards CckA (Figure 1B).

### Disruption of DivL-DivK∼P Binding Impacts Cell Fitness

**Figure 7:**
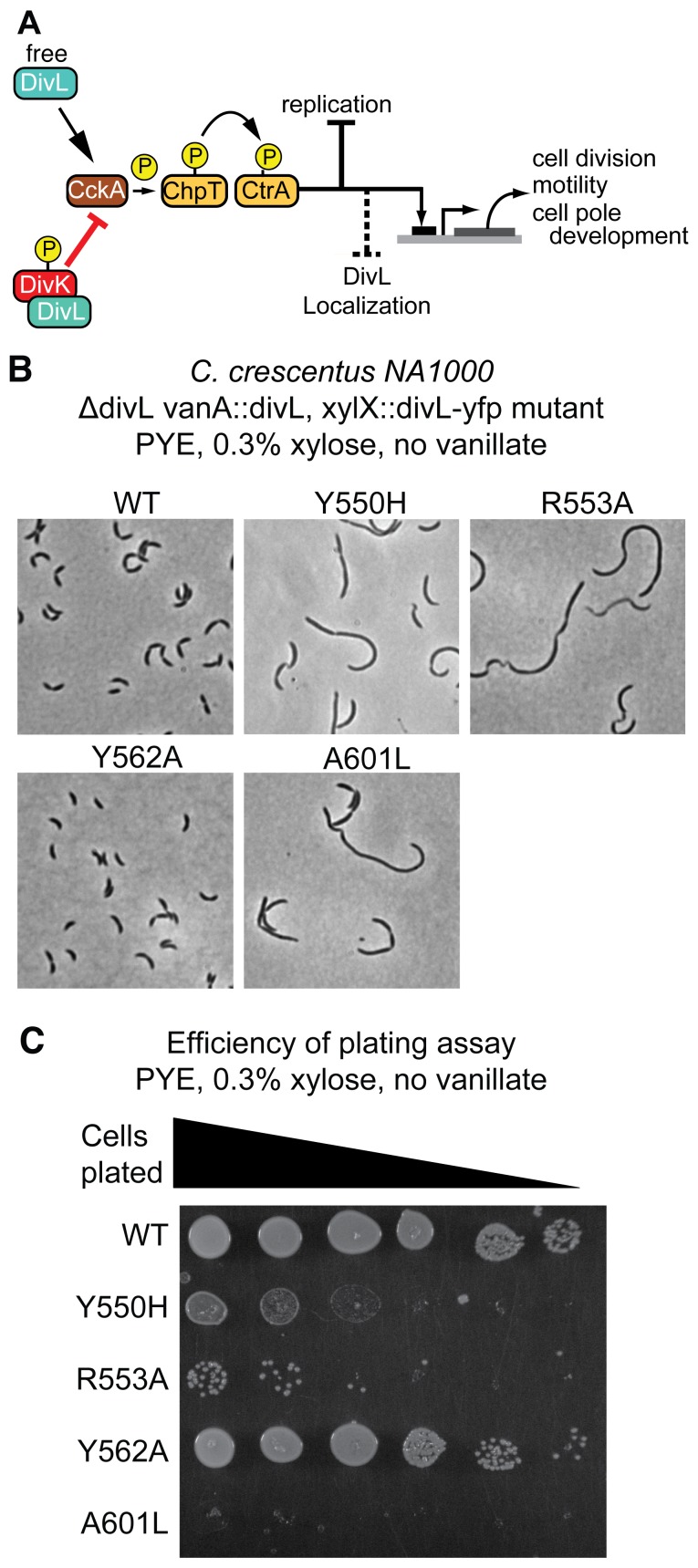
Impact of DivL point mutations on cell fitness. (A) Genetic model describing the functional consequences of DivL-DivK∼P binding on *Caulobacter* growth and development. Critically over-active CtrA impacts cell division, motility, and DivL subcellular localization. (B) Morphology assay of *Caulobacter* strains grown in PYE indicates that Y550H, R553A and A601L display cell filamentation and cell division defects, while the Y562A variant is similar in morphology to a wild-type strain. (C) An efficiency of plating assay indicates a reduction in growth rates of Y550H, R553A, and A601L, while Y562A displayed a mild reduction in growth rate relative to wild-type.

Previous genetic experiments have indicated that excess CtrA∼P can impact cell growth, motility, replication, cell division [7], and DivL localization (Figure 7A) [39]. Since the DivL-DivK∼P interaction is known to result in inhibition of the CckA phosphorelay [13], we predicted that the DivL mutations characterized here, which impact binding of DivK∼P to DivL, should consequently reduce cell fitness through dysregulation of CtrA signaling. Therefore, we generated translational fusions of each *divL* mutant allele described above to the coding region for eYFP and introduced the fusion constructs into the *Caulobacter* genome at the *xylX* locus under control of the xylose inducible P_xylX_ promoter [40]. The strain background employed in each experiment contained a previously constructed *divL* depletion construct in which the wild-type *divL* ORF was placed under the control of the vanillate-inducible P*_vanA_* promoter at the chromosomal *vanA* locus [16]. In the resulting merodiploid strains, wild-type and mutant *divL* alleles could be conditionally expressed through the addition of vanillate or xylose, respectively, to the growth medium.

We expressed multiple *divL-eyfp* mutant alleles (Y550H, R553A, and A601L) grown in peptone yeast extract (PYE) media supplemented with xylose. Cell lengths were significantly longer in cells expressing Y550H, R553A, and A601L, while Y562A exhibited no obvious cell morphology defects (Figure 7B). Plating efficiency assays indicated growth defects ranging from very mild (Y562A) or moderate (Y550H) to severe in A601L cells (Figure 7C). Interestingly, the R553A mutant was similar to Y550H on PYE plates with no xylose, but additional expression of the mutant by addition of xylose further inhibited the growth of the R553A mutant specifically, suggesting a dominant negative effect (compare Figures 7C with S9). Monitoring growth in liquid culture by optical density also revealed additional xylose induced expression of R553A was toxic (Figure S9). In a mixed population of cells grown in minimal media, we observe that 42% of cells expressing DivL-eYFP exhibit an observable monopolar DivL population (Figure S9), whereas only 20% of cells containing DivL-DivK∼P binding mutations (Y550H, R553A, and A601L) had an observable monopolar focus and the Y562A DivL mutant had a mild reduction (32%) in the proportion of cells with monopolar DivL (Figure S9). Overall, these results demonstrate that two DivL-DivK∼P binding mutants, A601L and R553A, have severe impacts on cell fitness, while the Y562A mutation has a relatively mild effect even though DivK∼P binding is similarly affected by that substitution *in vitro*. Intriguingly, the strain containing the Y550H mutation exhibits clear growth and morphology defects despite the fact that DivK∼P binding to DivL is not disrupted in that background, suggesting that Y550H perturbs the activity of DivL in a distinct manner.

## Discussion

### Transmitter Core Converted to a Sensory Module

Our structural and biochemical analysis showed that DivL lacks both kinase and phosphatase activity but still retains its ability to dimerize and bind DivK∼P specifically. The DivL mutant Y550H supports viability but impacts cell morphology, doubling time, and the subcellular localization of DivL. These defects suggest that the replacement of histidine by tyrosine is critical for optimal DivL function. We observed that DivL Y550H bound to DivK∼P with similar affinity as the wild type, indicating that the tyrosine substitution neither enhances nor diminishes DivK∼P binding. The Y550H mutation did not rescue autonomous autokinase activity *in vitro*, however, DivL(Y550H) autokinase activity can be stimulated by DivK∼P in the presence of ATP (Figure 6D). Thus, the H to Y substitution in DivL does not perturb HK-RR binding but abrogates kinase activity. From these studies, we propose that the DivL pseudokinase has repurposed its catalytic HK fold to function primarily as a sensory module for a phosphorylated RR. Loss of catalytic function ensures orthogonal sensor function while preventing unwanted signal modification (i.e., inter-conversion of DivK and DivK∼P) that would compromise asymmetric cell division.

### A Role for Histidine Kinase PAS Domains in Phosphospecific RR Recognition

Previous studies of two-component systems have highlighted the critical role of the RR docking module in determining HK-RR phosphotransfer specificity [21],[41]–[45]. Consistent with these studies, we identified four point mutations within the RR docking module that disrupt DivK∼P binding to DivL (Figure 4). Thus, our results support the notion that DivL binds DivK∼P in a conserved manner similar to a canonical HK-RR pair [23],[41]–[44]. However, a key question remains: how does DivL specifically recognize DivK∼P? The tyrosine or histidine side chain at position 550 could contribute to the specific recognition of the phosphoryl group on DivK directly, by forming a hydrogen bond. However, we do note that Y550F has been previously shown to specifically bind phosphorylated DivK over unphosphorylated DivK [13]. Furthermore, the DivL RR docking surface, which includes Tyr550, may be tuned to specifically recognize structural changes in DivK that are induced by phosphorylation. Here, we observed that the PAS sensor domain plays an unexpected role in phosphospecific discrimination of DivK∼P over DivK (Figure 5). One possible role of the PAS sensor domain could be to directly sterically block unphosphorylated DivK binding while permitting DivK∼P binding, or perhaps more likely the DivL PAS domains could indirectly reconfigure the DivK binding site to promote phosphospecific binding. The PAS domain's role in phosphospecific RR binding is consistent with the finding that optimal WalK [46] and ThkA [25] phosphatase activity requires a PAS module. More generally, it appears that PAS sensor domains may regulate kinase/phosphatase activity by influencing the phosphospecific binding preference of an HK for its cognate RR.

### Models for the DivL Cell-Fate Switch

Several recent biochemical studies of HKs have revealed that HKs transmit signaling information over a long-range through large-scale conformational changes in order to regulate activity. DHp conformational rearrangements, such as “twisting” (cogwheeling or rotation) and “bending” (kinking), can produce unique functional states that are crucial for HK regulation [24],[26]–[28],[32],[47]–[50]. Our rigid-body structural analysis of DivL suggests that interhelical bending could alter the relative position of domains that regulate DivL function. Indeed, our binding studies indicate that a mutation at the interface between two rigid bodies (A601L), disrupts DivK∼P binding in a PAS-domain-dependent manner (Figure 5C). Furthermore, the A601L mutation causes a more severe phenotype than does the direct RR docking mutation Y562A. This suggests that A601L may be pleiotropic, perhaps simultaneously affecting upstream (i.e., DivK∼P binding) and downstream events (i.e., CckA inhibition). Residue Arg553 may also serve a switch-like role triggering conformational changes upon DivK∼P binding by interacting with DivK∼P in the complexed state, and in the uncomplexed state interacting with the output-helix residue Asp595 (Figure 5). Taken together, this model may explain why A601L and R553A have stronger impacts on cell fitness than the Y562A variant. Additionally, DivK∼P-dependent rescue of DivL autokinase activity in the presence of Y550H suggests that DivK∼P binding triggers conformational changes in DivL that are necessary for ATP binding and autokinase activity.

**Figure 8:**
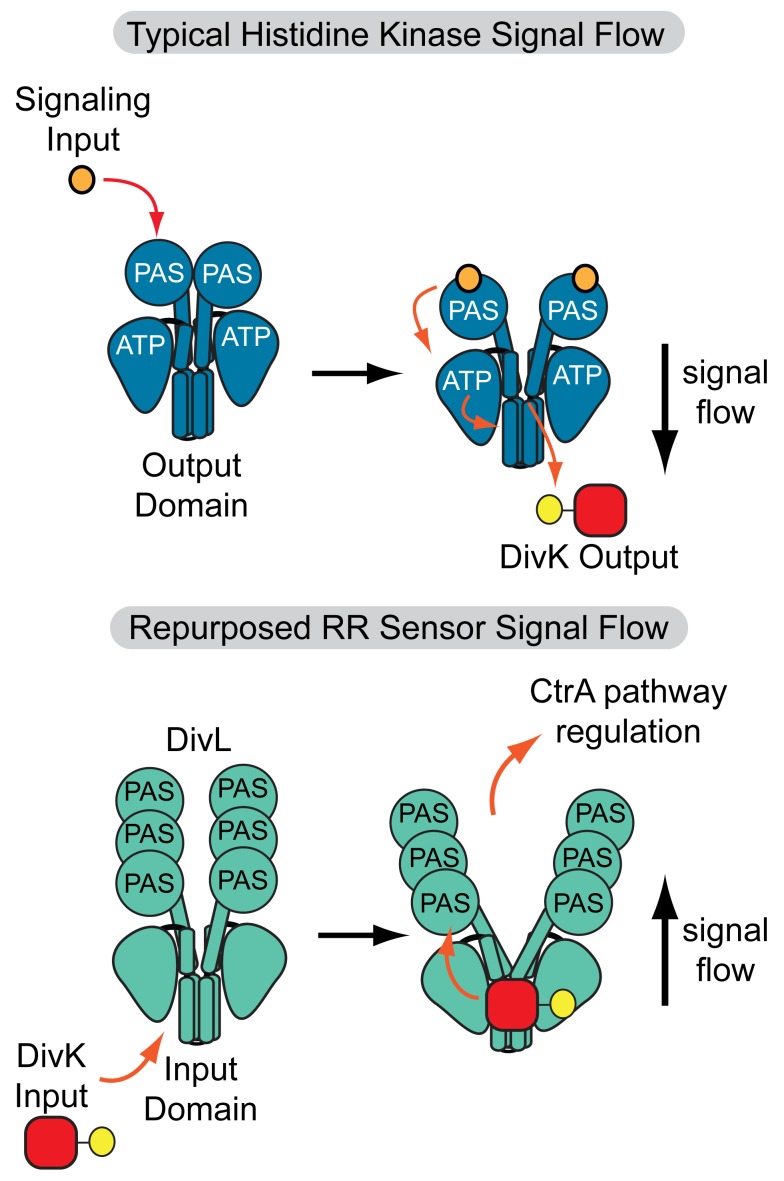
Model for a repurposed pseudokinase that functions as a sensor for a phosphorylated RR. In conventional two-component signaling systems, an HK receives a signal in the sensor region and ultimately promotes phosphorylation of a response regulator. In contrast, DivL repurposes its RR docking surface and canonical output domain as a sensory module for DivK∼P. Upon binding DivK∼P, DivL causes the repression of the CtrA differentiation pathway, potentially through conformational remodeling of the DivL PAS sensor domains.

Thus, we propose a model in which the interaction between Tyr550 and the phosphorylation site of DivK∼P induces bending in the α1 helix of DHp, which is then propagated to the rest of the structure via rigid-body movements. As a result of the conformational change, the CA and PAS domains of DivL may re-position to allow formation of additional contacts with bound DivK∼P. In the absence of catalytic functions, we suggest that DivL exploits the underlying large-scale conformational re-arrangements of the HK-fold to function as a DivK∼P-modulated two-state molecular switch (Figure 8). Asymmetric dimers have been proposed to represent important activation intermediates [26]–[29],[51], and it is conceivable that DivL undergoes a symmetric-asymmetric conformational switch upon DivK∼P binding. However, the mechanism by which DivK∼P-dependent conformational changes in DivL impact the CckA-ChpT-CtrA phosphorelay [12],[13],[15],[39],[52] remains unclear. It is plausible that DivL interacts directly or indirectly with CckA in the multi-protein polar complex, thus allowing the structural changes in DivL induced by DivK∼P to propagate to changes in CckA activity. Future experiments will focus on dissecting DivL's essential regulation of the CckA-ChpT-CtrA pathway.

### Broadening the Wiring Possibilities of Two-Component Systems

A common bacterial signaling strategy is to wire two-component systems as simple orthogonal linear signaling arrays to limit cross-talk and maintain the integrity of signaling information [43],[44]. Phosphorelay pathways offer a way to branch signaling pathways in order to enable multiple signaling inputs [30] or outputs [45],[53]. Signaling directionality in the two-component systems from a kinase to a response regulator apparently limits bacterial signal processing capacity relative to more complex eukaryotic systems. Here, we demonstrated an exception to this paradigm in which loss of HK catalytic activity repurposes an output module as a phosphospecific RR input sensor, thereby reversing the flow of information observed in conventional HK-RR systems. This reversal allows DivL to couple two distinct signaling pathways by serving as an “output” for a compartment sensing pathway and an “input” for the differentiation phosphorelay (Figure 8). The extent to which nature utilizes this reversed signaling warrants further investigation. For example, it could allow transmission cytosolic stress signals to the periplasm via membrane-bound HKs. The presence of pseudokinases in two-component signaling systems therefore offers a general mechanism for coupling signaling pathways, reminiscent of complex eukaryotic signaling networks.

## Materials and Methods

### Construction of Plasmids and Strains

DNA primers, plasmids, plasmid construction methods, and bacteria strains used in this study are listed in Tables S3, Table S4, Table S5, and Table S6, and detailed cloning methods are included in Text S2. The domain architecture of DivL was annotated using the HHpred structure prediction algorithm [54]. For the DHp-CA construct we retained a significant portion of the N-terminal input helix that connects the sensor domains to the DHp domain to generate DivL(523–769). Constructs with N-terminal start codons at: 411 containing one PAS domain, 281 containing two PAS domains, and 152 containing three PAS domains. DivL residues 1–53, which were predicted to form a transmembrane helix followed by a coiled-coil region, were not included to improve *in vitro* solubility.

### Protein Expression and Purification

Protein expression of all DivL variants, DivJ, PleC, and DivK followed the same protocol and is described in detail below for the crystallization construct DivL(523–769). Protein expression of ChpT, CckA, and CckA H322A were performed as described previously [45]. Plasmid pWSC8 was then transformed into chemically competent Rosetta (DE3) pLysS cells, and plated onto selective LB media (30 µg/ml chloramphenicol and 50 µg/ml kanamycin plates), and grown overnight at 37°C. From a single colony, an overnight 25 ml LB (chloramphenicol: 20 µg/ml; kanamycin: 30 µg/ml) culture was inoculated and grown to saturation overnight. From this saturated culture a 2L LB culture was inoculated and grown to mid-log phase (∼0.6 OD). Expression of DivL(523–769) was induced with 333 µM isopropyl-β-D-thiogalactopyranoside (IPTG) for 4 hours at 25°C. The cells were harvested in an ultracentrifuge at 4°C, 30 minutes, 5,353 *g*. The resulting pellet was resuspended in 40 ml 50 mM HEPES pH 7.9, 0.5 M NaCl and centrifuged at 3,700 g at 4°C for 20 to yield a cell pellet stored at −80°C.

Cells were thawed on ice and resuspended in 40 ml of lysis buffer (50 mM HEPES pH 8.0, 0.5 M KCl, 1 mM DTT, 25 mM imidazole, and 200 U of benzonase nuclease) supplemented with SIGMAFAST™ protease inhibitor tablets (Sigma). The cell suspension was lysed with three passes through a French Press at 20,000 psi. Insoluble cell debris was pelleted via centrifugation (27,216 *g*, 45 min at 4°C). The resulting supernatant was incubated with 2 ml of a 50% slurry of Ni-NTA agarose at 4°C for 1 hour. The Ni-NTA agarose was pelleted and washed with 150 ml of Ni-NTA wash buffer (50 mM HEPES [pH 7.9], 0.5 M KCl, 1 mM DTT, and 25 mM imidazole). Then His6-DivL(523–769) was eluted from the agarose with Ni-NTA elution buffer (50 mM HEPES [pH 8.0], 0.5 M KCl, 1 mM DTT, and 250 mM imidazole) and concentrated to a 2 ml volume using Amicon Centrifugal Filter Units (10 kDa cutoff). This concentrated elution was loaded onto a Hi-Prep 16/60 Sephadex S-200 gel filtration column (GE Healthcare), eluted in kinase buffer (50 mM HEPESáKOH [pH 8.0], 200 mM KCl, 0.1 mM EDTA, 10% (v/v) glycerol, and 1 mM DTT). The eluted His_6_-DivL(523–769) was then concentrated to ∼15 mg/ml using amicon centrifugal filter units, aliquoted and frozen in liquid nitrogen for crystallization trials. The concentration of His_6_-DivL(523–769) was determined using ε_280_ of 8,605 M^−1^cm^−1^.

MBP-DivK expression and purification followed the same protocol, however after elution from the Ni-NTA agarose TEV protease was used to cleave off the His-MBP-TEV site tag of DivK. Briefly, His_6_-MBP-DivK was diluted to 2 mg/ml and mixed with His_6_-TEV protease and the solution was dialyzed overnight at 4°C in two 12 ml Slide-A-Lyzer 10 k MWCO dialysis cassettes against 2L dialysis buffer (20 mM HEPESáKOH [pH 8.0], 100 mM KCl, 0.5 mM EDTA, 10% glycerol, 1 mM DTT). The following day, the His6-tagged unreacted protein impurities were removed by subtractive Ni-NTA affinity purification with 6 ml of 50% slurry of Ni-NTA agarose affinity resin (5Prime) equilibrated in dialysis buffer; the eluate containing cleaved DivK was collected.

### Crystallization

The initial crystallization conditions for DivL were obtained using the sparse matrix screening method (Hampton Research). The conditions were subsequently optimized manually to improve the quality of the crystals. The crystals used for structure solution were obtained using the hanging-drop vapor diffusion method at 22.5°C. The reservoir well contained 500 µl 0.1 M HEPES (pH 7.5) and 18% PEG 10000, while the drop contained 1.2 µl of DivL (concentration 5.8 mg/ml) mixed with 1.2 µl of the reservoir solution. For cryoprotection, the crystals were gradually transferred to a series of reservoir solutions containing incrementally higher concentration of PEG 4000, up to a final concentration of 30% prior to flash freezing in liquid nitrogen. The data were indexed and processed in the trigonal space group P3_1_21 with unit cell dimensions of *a* = 69.5 Å and *c* = 194.4 Å.

To obtain heavy atom derivatives, crystals were soaked in cryo solution containing 10 mM KAu(CN)_2_ for 10 min. For co-crystallization with nucleotides, MgCl_2_ and each nucleotide (ATP, ADP, AMP-PNP, AMP-PSP, and TNP-ATP) were added in ∼10 molar excess to the protein before crystallization. In addition, attempts were made to soak the native crystals in the presence of nucleotides and magnesium.

### Data Collection, Structure Determination, and Refinement

Native data and multiwavelength anomalous diffraction (MAD) data for the gold derivative, and data for crystals obtained in the presence of different nucleotides were collected at the SSRL Beamline 12-2. The datasets were collected at 100 K using a Pilatus 6M pixel array detector (Dectris). Each dataset was integrated using XDS and then scaled with the program XSCALE [55]. Gold sites were located with SHELXD [56] using the data corresponding to the peak wavelength (1.0397 Å) of the Au-MAD experiment. Phase refinement (FOM = 0.32 for three Au sites) and automatic model building were performed using autoSHARP [57] and BUCCANEER [58]. Model completion and refinement were performed with COOT [59] and BUSTER [60]. Additional CCP4 programs [61] were used to for data conversion and other calculations. Data reduction and refinement statistics are summarized in Table S2. Atomic coordinates and experimental structure factors for DivL at 2.5 Å resolution have been deposited in the PDB (http://www.rcsb.org) under accession code 4q20.

### Gel Filtration

A gel filtration standard (Bio-rad) containing thyroglobulin (bovine), γ-globulin (bovine), ovalbumin (chicken), myoglobulin (horse), and vitamin B12 were used to generate a molecular weight standard plot using a Superdex 200 10/300 GL column (GE Healthcare). A 2 mg/ml sample of His_6_-DivL(523–769) was loaded onto the column and eluted after 14.2 ml, corresponding to a molecular weight of 50±15 kDa (dimer = 57.4 kDa). Error bars for molecular weight estimated as the full width at half maximum (FWHM) for the eluting peak.

### Co-immunoprecipitation Assay

Immunoprecipitations were performed as previously described [62]. A 500 ml culture of wild type *Caulobacter* cells (strain LS101) and a strain carrying divL-m2 (LS4468) [16] were grown in PYE media at 28°C to mid-log phase. The cells were harvested in a centrifuge at 4°C, 15 min, and 7,800 rpm. Cells were then washed with CO-IP buffer (20 mM HEPES pH 7.5, 100 mM NaCl, 20% glycerol), and pelleted in a centrifuge at 4°C, 15 min, and 9,000 rpm. Cells were resuspended in 29.2 ml PBS (pH 6.8) containing 1% formaldehyde and allowed to cross-link at room temperature for 30 minutes. Cells were pelleted in a centrifuge at 4°C, 15 min, 9,000 rpm and then resuspended in 30 ml of CO-IP buffer supplemented with protease inhibitors. The pellet was snap frozen with liquid nitrogen and stored at −80°C. The cell suspension was thawed and lysed with three passes through a French Press at 20,000 psi. After lysis, 2 mM EDTA and 1% Triton X-100 were added and allowed to incubate on ice for 60 min. Insoluble cell debris was pelleted via centrifugation (9,000 rpm, 10 min at 4°C). Lysate was incubated with 50 µl of FLAG-M2 agarose (FLAGIPT-1 kit; Sigma) overnight on a nutator at 4°C. Beads were washed twice with co-IP buffer supplemented with 0.05% NP-40, then washed five times with wash buffer (50 mM Tris-HCl, 150 mM NaCl, protease inhibitors, 0.05% NP-40) and proteins were eluted by incubating with 3× FLAG peptides. Western blots were performed as previously described [62]. Antibody dilutions were as follows: α-FLAG (Sigma) 1∶1,000,) and α-DivK (1∶5,000). Films were scanned and processed with Adobe Photoshop.

### Fluorescence Polarization Assay

DivK was labeled at Cys-99 using thiol-reactive BODIPY FL N-aminoethyl malemide (Invitrogen). DivK was mixed together with 10-fold excess BODIPY FL N-aminoethyl malemide and allowed to react for 2 hours at room temperature, and unreacted dye was quenched with mercaptoethanol. BODIPY-DivK was purified via dialysis to remove unreacted fluorescent dye. BODIPY-DivK∼P was generated by mixing 250 nM DivJ with 1 mM ATP and 5 mM MgCl_2_ and incubated for 40 min. Mock binding assays were done in the presence of [γ-^32^P]ATP, and under these reaction conditions scintillation counting combined with Bradford assay estimate >75% of DivK was in the phosphorylated state. Upon phosphorylation of BODIPY-DivK by 250 nM DivJ no change in fluorescence polarization was observed. This finding indicates that BODIPY-DivK∼P was likely a monomer under reducing buffer conditions (1 mM DTT) and that no significant DivJ was bound to DivK.

For binding assays using unphosphorylated DivK, 1 mM non-hydrolyzable AMP-PNP and 5 mM MgCl_2_ were included in the buffer. BODIPY-DivK∼P or BODIPY-DivK was then incubated with varying kinase concentrations for 45 minutes to reach binding equilibrium. Fluorescent DivK was excited at 470 nm and emission polarization was measured at 530 nm in a Molecular Devices SpectraMax M5 plate reader. Fluorescent polarization measurements were performed in triplicates, and three independent trials were averaged with error bars representing the standard deviation. We extensively screened buffer conditions to optimize DivL-DivK binding by exploring KCl concentration, % glycerol, and pH. Of these parameters, DivL-DivK binding was most sensitive to KCl concentration and low KCl (50 mM) promoted the DivL-DivK interaction (Figure S1). Interestingly, ATP appeared to promote the interaction between DivL-DivK and DivL-DivK∼P (Figure S1).

### Coupled-Enzyme Activity Assay

ATPase activity of DivL constructs was measured using a coupled-enzyme assay [63],[64]. Proteins were mixed in kinase buffer supplemented with 1 mM ATP, 10 mM MgCl_2_, 1 mM phosphoenolpyruvate, 0.2 mM NADH, 2 units of pyruvate kinase, and 6.6 units of lactate dehydrogenase. Reactions were performed in triplicate in 200 µl volumes and loaded into a clear, polystyrene 96-well plate. Reactions were initiated by the addition of protein, and absorbance at 340 nm was recorded every 10 seconds for a 30 minute period. The slope of a stable linear absorbance decay was measured to calculate ATP hydrolysis rates, using a NADH Kpath value of 3,248 OD M^−1^ [63]. Background rates of ATP hydrolysis and NADH oxidation were measured and subtracted from observed ATP hydrolysis rates of all DivL constructs.

### Autophosphorylation Reactions

CckA(70–691) and DivL(152–769) constructs at 5 µM were incubated for one hour at room temperature in kinase buffer supplemented with 0.5 mM ATP, 0.167 µCi/µl [γ-^32^P]ATP, and 5 mM MgCl_2_ in a total reaction volume of 50 µl. Reactions were stopped by the addition of 2× Laemmli sample buffer, then loaded onto 12% Tris-HCl gels for electrophoresis. The radioactivity in wet gels was recorded on phosphor storage plate for 3 h, and then imaged on a Typhoon fluorescence imager (Molecular Dynamics). Quantitation of band intensities was measured using ImageJ and band intensities for three individual experiments were averaged. The mean intensity and standard deviation were plotted using Prism 6 software (GraphPad).

### DivK∼P Dephosphorylation Assays

For DivK∼P phosphatase assays, DivK was incubated with 250 nM His-DivJ, 1 mM ATP, and 5 mM MgCl_2_ on Ni-NTA agarose resin for 40 min. DivK∼P was eluted and purified away from His-DivJ, followed by a second round of Ni-NTA purification. Purified DivK∼P was then incubated with excess hexokinase and glucose for 10 minutes to convert remaining ATP into ADP. Purified DivK∼P was then incubated with either PleC, DivL(152–769), or DivL(152–769) Y550H for varying amounts of time. Reactions were quenched with 4× SDS-PAGE sample buffer. Gels were processed as described above in the autophosphorylation section. The mean intensity and standard deviation from three independent experiments were plotted using Prism 6 software (GraphPad).

### TNP-ATP Binding Assay

A solution of 1 µM TNP-ATP [34] was compared with a solution of 2 µM TNP-ATP with 10 µM DivL(523–769) in 50 mM HEPES×KOH (pH 8.0), 200 mM KCl, 0.1 mM EDTA, 10% (v/v) glycerol. Solutions were allowed to reach equilibrium for 30 min at room temperature, prior to fluorescence measurements. An emission profile was collected over a wavelength range of 475–750 nm. Presented curves in Figure S7 are average values of three replicates.

### Filter Binding Assays

Filter binding assays were designed to capture transiently bound [γ-^32^P] ATP [65],[66]. Solutions of DivL(523–769), DivL(523–769) Y550A, CckA (70–691) H322A, ChpT, and BSA at 10 µM protein concentration was mixed with 27.5 fmol of [γ-^32^P] ATP in kinase buffer at room temperature. Empirically, we found a 30-minute period allowed adequate time for equilibration, while minimizing hydrolysis and release of ATP. Solutions were passed over a nitrocellulose membrane filter and washed three times with 1 ml of kinase buffer. Bradford assays indicated >95% of protein remained bound to the nitrocellulose. Subsequently, nitrocellulose filters were submerged into scintillation fluid and scintillation counted to quantify bound [γ-^32^P] ATP. [γ-^32^P] ATP standard solutions were used to determine the specific activity and convert scintillation counts in fmol bound to the nitrocellulose. [γ-^32^P] ATP only solutions were passed through nitrocellulose membranes to quantify non-specific binding.

### Growth and Morphology Assays of DivL-DivK Binding Mutants

 Each of the pXYFPC-*divL* mutant strains were transformed into *Caulobacter* NA1000 strain AA871 that is ΔdivL vanA::*divL*. In these strains the impact of *divL* mutant expression was evaluated by removal of vanillate from media to deplete wild-type *divL* from cells, and addition of xylose to express *divL-yfp* mutants. Mutant *divL* genes were examined in a strain where the native copy of *divL* was deleted, the WT *divL* placed under vanillate induction at the *vanA* locus, and a mutant *divL-yfp* was placed at the xylose-inducible *xylX* locus of the chromosome. Cells were grown for 24 h in PYE containing no vanillate and no xylose. Cells were then diluted in fresh, xylose-containing media to optical density at 600 nm (OD-600) of 0.02 (diluted to approximately 0.06 for efficiency of plating assays). The cells were then allowed to grow for several hours in the xylose until either being plated or imaged. For efficiency of plating assays, cells grew for either 0 or 6 hours after addition of xylose. Cells were spotted on PYE-agar plates in 3 µl volumes. The first, leftmost spot was spotted from the 0.06 OD-600 culture, and subsequent dilutions were made serially in 5-fold steps. The xylose content of the plates was varied from 0 to 0.3% w/v, and cells were plated from liquid media containing an equal amount of xylose. The plates were then grown at 28°C for 2 days and subsequently imaged. For liquid culture growth curves, we measured the OD-600 every 1–2 hours for 10 hours or until the culture reached saturation.

### Microscopy

ΔdivL vanA::divL xylX::divL-yfp WT, Y550H, R553A, Y562A, and A601L strains (WSC399, WSC308, WSC310, WSC312, and WSC314) were cultured overnight in M2G supplemented with 5 µM vanillate and sub-cultured into fresh media containing 0.03% xylose for 6 hours or until they reached an OD600 of 0.4 for *divL* localization assays. Cell suspensions were then dried onto agarose pads (1.5% agarose in M2G) and imaged on a Leica DM 6000 B microscope with a HCX PL APO 100°—/1.40 Oil PH3 CS objective, Hamamatsu EM-CCD 15 C9100 camera and Metamorph (Molecular Devices). Both phase-contrast and fluorescence images were recorded. Images were processed in Adobe Photoshop, and quantitative analysis of images was performed using ImageJ's cell-counter. For morphology assays, the cells were grown overnight in PYE and switched into fresh PYE with xylose. After 6 hours of xylose induction, cells were placed on a PYE-agar pad for imaging. Images were processed in Adobe Photoshop.

## Supporting Information

Figure S1 **Interaction between DivK and DivL, DivJ, or PleC.** (A) Multiple sequence alignment of the DHp domains of three known DivK binding partners, DivL, DivJ, and PleC. The predicted residues involved in the interactions with DivK are highlighted at the bottom (orange, hydrophobic contacts; blue, hydrogen bonds involving side-chains). (B) *In-vivo* co-immunoprecipitation of DivK with DivL-m2 from *Caulobacter* lysates. (C–E) DivK (black) and DivK∼P (red) binding curves for DivJ, PleC, and DivL using a fluorescence polarization assay. Influence of buffer conditions upon the interaction between DivL-DivK∼P using the DivL(523–769) construct by varying the concentration of (F) KCl, (G) pH, and (H) % glycerol. (I) Examination of the impact of ATP binding upon the interaction of DivL-DivK (unphosphorylated). Numerical data used to generate manuscript graphs or histograms can be found in Table S1.
(TIF)

Figure S2 **Sequence conservation of DivL orthologs mapped on the DivL structure.** (A) The sequence of the HK region of DivL mapped with the secondary structures. The sequence is colored in gradient by degrees of sequence conservation, calculated from top 40 DivL homologs with sequence identity between 35% and 90%, from not conserved (white background) to strictly conserved (black background). The relative solvent accessibility of each residue (ratio of solvent accessible surfaces of each residue in the structure and in solution) is shown by blue boxes above the sequence. The residues that are near the DHp-CA interface in the structure shown in Figure S3 are marked by orange dots below the sequence. The five sequence motifs (H, N, G1, F, and G2) defined by Parkinson and Kofoid [67] are marked at the bottom. (B) Mapping of sequence conservation as a grayscale with black (most conserved), white (non-conserved) onto the DivL dimer, shown in cartoon (left) and surface (right) representations.
(TIF)

Figure S3 **Structure comparisons, domain interface between DHp and CA, and B-value distribution of DivL.** (A) Structural comparison of DivL to HK853 (phosphotransfer/phosphatase state, PDB code 3dge [23]), ThkA (PDB code 3a0r[25]), and KinB (PDB code 3d36 [31]). The highly conserved CA domains in all structures are shown in the same orientation. Residues at the phosphorylation site and ADP are shown in sticks. (B) Stereoview of the “RR docking modules” in HKs (HK853 and KinB) and ChpT. The structures were superposed using a stretch of 15 residues starting from the phosphorylation site. (C) Stereoview of the substructures that include 〈2C and the CA domain in DivL-A, DivL-B, HK853, QseC (PDB code 3jz3), and ThkA. (D) Overview of the DHp-CA interface in the DivL dimer. The residues near the domain interface are highlighted in orange. (E) A close-up view of the DHp-CA interactions in DivL-A. (F–G) Distribution of B-values in the DivL structure, shown in cartoon (F) and surface (G) representations. The B-values are colored in a gradient from low (blue) to high (red).
(TIF)

Figure S4 **A model of DivL-DivK interaction.** (A) A model of DivL and DivK interaction, shown in stereoview. The model is based on the HK853 complex with its cognate RD RR468 (PDB code 3dge). DivK (PDB code 1mav) is shown in red, and DivL in violet and cyan. The phosphorylatable residue Asp53 of DivK and Tyr550 of DivL are shown as sticks. (B) DivL-A/DivK (violet/red) and DivL-B/DivK (cyan/red) are superimposed using the “RR docking modules” and DivK, indicating that structural changes in DivL can impact its interactions with DivK. Asp53, Gln55, and Asp90 of DivK and Tyr550 of DivL are shown as sticks.
(TIF)

Figure S5 **Binding curves for DivL(152–769) binding mutants to DivK∼P (red curves) and compared to wild-type DivL (black curves) for the following point mutations: (A) Y550H, (B) R553A, (C) T557N, (D) Y562A, (E) H579E, and (F) A601L.** Numerical data used to generate manuscript graphs or histograms can be found in Table S1.
(TIF)

Figure S6 **DivL has no kinase activity** ***in vitro*** **.** (A) DivL constructs used in the assay. (B) Comparison of kinase activities of various DivL constructs relative to the CckA kinase controls. NADH coupled enzyme assay compares the NADH consumption rate at 5 µM concentration for each protein. (C) Comparison of autophosphorylation reactions of 5 µM CckA(70–691) or DivL(152–769) in kinase buffer supplemented with [γ-^32^P] ATP and 5 mM MgCl_2_. Numerical data used to generate manuscript graphs or histograms can be found in Table S1.
(TIF)

Figure S7 **Nucleotide-binding assays.** (A) Binding of 1 µM TNP-ATP (red) compared with 10 µM DivL and 1 µM TNP-ATP (black). (B) Titration of 2–40 µM DivL into 0.5 µM TNP-ATP for binding constant estimate of 57±8 µM, Bmax of 18, and R^2^ = 0.9976. (C) The impact of MgCl_2_ on the binding of TNP-ATP to DivL. Fluorescence emission profile of 5 µM TNP-ATP (black), 5 µM TNP-ATP+5 µM DivL (blue), 5 µM TNP-ATP+5 µM DivL+5 mM MgCl_2_ (red). (D) Filter binding assay indicating percent of 27.5 fmol of [γ-^32^P] ATP bound to 10 µM DivL(523–769), 10 µM DivL(523–769) Y550A, CckA(70–691) H322A, ChpT, and BSA. (F–G) ATP binding site in DivL. (E) Stereoview of the 2Fo-Fc electron density map near the nucleotide binding region of the ADP and Mg soaked crystal, contoured at 1.0 σ (blue). The refined model is shown as a Cα trace (magenta). (F) Stereoview of the ATP binding site of DivL-A (violet), DivL-B (cyan), and KinB (gray). All structures are superposed together using the CA domains. ADP and Mg molecules from KinB are shown. The residues near the ADP molecule are shown in sticks. (G) The ATP binding site on the DivL-A (left) and DivL-B (right). Each monomer is colored in violet and cyan. An ADP molecule, shown as sticks, was modeled into the ATP binding site based on the structure of KinB. The ATP lid is colored in red. Tyr550 is colored in yellow. An ADP molecule, shown in spheres in the same scale, highlights the size of ADP compared to the opening for ATP entry. Numerical data used to generate manuscript graphs or histograms can be found in Table S1.
(TIF)

Figure S8 **DivL(152–769) Y550H accumulates phosphate in a DivK∼P and ATP-dependent manner.** Comparison of reaction mixtures of DivL(152–769) Y550H with phosphorylated DivK∼P in the presence and absence of ATP.
(TIF)

Figure S9 **Effects on** ***Caulobacter*** **growth and DivL localization by site-directed mutations disrupting DivL-DivK interaction.** (A) Growth curves of strains containing *divL* point mutants. Growth was measured by optical density for 10 hours or until culture saturation. Mutants were binned depending on their location on the RR binding surface or based on allostery involving the input-output helix of DivL. Growth curves were consistent across triplicate measurements, and one representative curve is shown for each mutant grown with and without xylose. Curves were fitted to a single exponential model. Inset: cartoon of DivL depicting the approximate location of each mutant. (B) Cell growth assayed by efficiency of plating. Prior to plating, cells were grown in PYE liquid media to OD-600 of 0.2 in the absence of xylose for 6 h. The culture was diluted 10-fold for the left-most inoculation, and 5-fold for subsequent dilutions. Plating was performed in duplicate using no xylose concentrations or vanillate. A representative plate is shown. (C–E) DivL-DivK binding mutants impact DivL-yfp subcellular localization. *divL-yfp* mutant strains were grown in M2G supplemented with 0.003% xylose. (C) Fluorescent images of *divL-yfp* mutant strains were grown in M2G supplemented with 0.003% xylose for DivL-DivK binding mutants: Y550H, R553A, Y562A, and A601L relative to wild-type *divL-yfp*. (D) Cell population analysis characterizing the percentage of cells with an observable monopolar focus. (E) Single-cell analysis of the fraction of DivL-yfp signal localized at the cell pole in cells with an observable monopolar focus. Numerical data used to generate manuscript graphs or histograms can be found in Table S1.
(TIF)

Table S1 **Numerical data used to generate manuscript graphs or histograms.**
(XLSX)

Table S2 **Data collection and refinement statistics.**
(DOCX)

Table S3 **DNA oligos used in this study.**
(DOCX)

Table S4 **Plasmids and strains used in this study.**
(DOCX)

Table S5 **Gibson cloning strategy to generate His-DivL(152–769) point mutants into pTEV5 for protein expression.**
(DOCX)

Table S6 **Gibson cloning strategy to generate DivL-yfp point mutants into the pXYFPC-1 vector that is designed to integrate at the xylose chromosomal locus using the Gibson DNA assembly method.**
(DOCX)

Table S7 **Accession numbers for genes and proteins.**
(DOCX)

Text S1 **Supplementary discussion of the DivL structure.**
(DOCX)

Text S2 **Detailed methods for Construction of plasmids and strains.**
(DOCX)

Movie S1 **A movie clip of a DivL dimer that illustrates “morphing” between the two observed DivL monomer conformations.** The monomeric subunits DivL-A and DivL-B are colored in violet and cyan, respectively. The ATP lid in each monomeric subunit is colored red, and Tyr550 is shown in yellow sticks.
(MOV)

Movie S2 **A movie clip of a DivL monomeric subunit (shown in cyan) that illustrates “morphing” between the two observed DivL monomer conformations.** The ATP lid is shown in red and Tyr550 is shown in magenta sticks.
(MOV)

## Abbreviations

DHp: dimerization and histidine phosphotransfer
HK: histidine kinase
RR: response regulator
