# Supplementary figures and images for: Cell Fate Regulation Governed by a Repurposed Bacterial Histidine Kinase
Source: PLoS Biol. 2014 Oct 28;12(10):e1001979. doi: 10.1371/journal.pbio.1001979 (PMC4211667; doi:10.1371/journal.pbio.1001979)

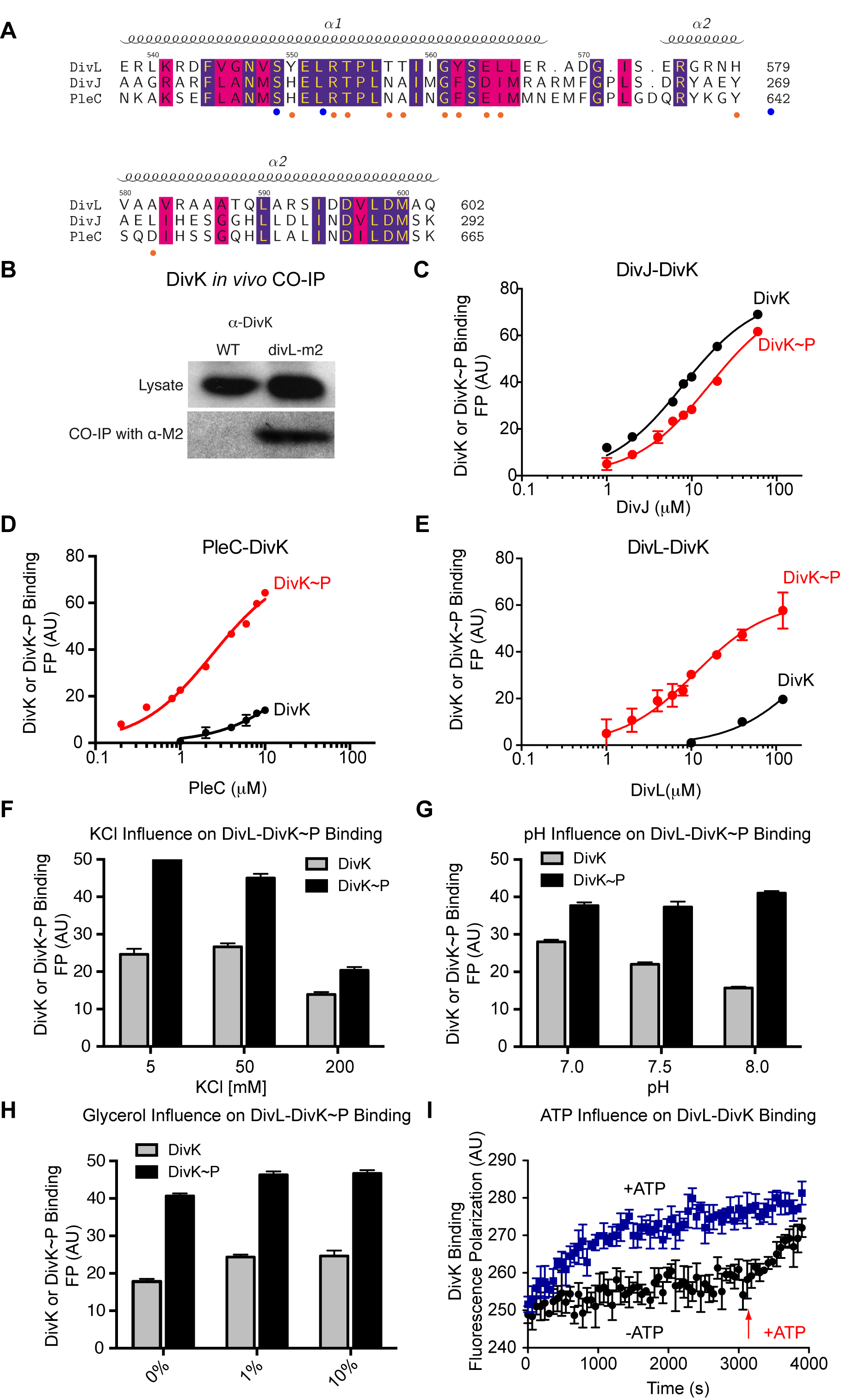

Supplement: Figure S1 — Interaction between DivK and DivL, DivJ, or PleC. (A) Multiple sequence alignment of the DHp domains of three known DivK binding partners, DivL, DivJ, and PleC. The predicted residues involved in the interactions with DivK are highlighted at the bottom (orange, hydrophobic contacts; blue, hydrogen bonds involving side-chains). (B) In-vivo co-immunoprecipitation of DivK with DivL-m2 from Caulobacter lysates. (C–E) DivK (black) and DivK∼P (red) binding curves for DivJ, PleC, and DivL using a fluorescence polarization assay. Influence of buffer conditions upon the interaction between DivL-DivK∼P using the DivL(523–769) construct by varying the concentration of (F) KCl, (G) pH, and (H) % glycerol. (I) Examination of the impact of ATP binding upon the interaction of DivL-DivK (unphosphorylated). Numerical data used to generate manuscript graphs or histograms can be found in Table S1. (TIF) [file pbio.1001979.s001.tif]

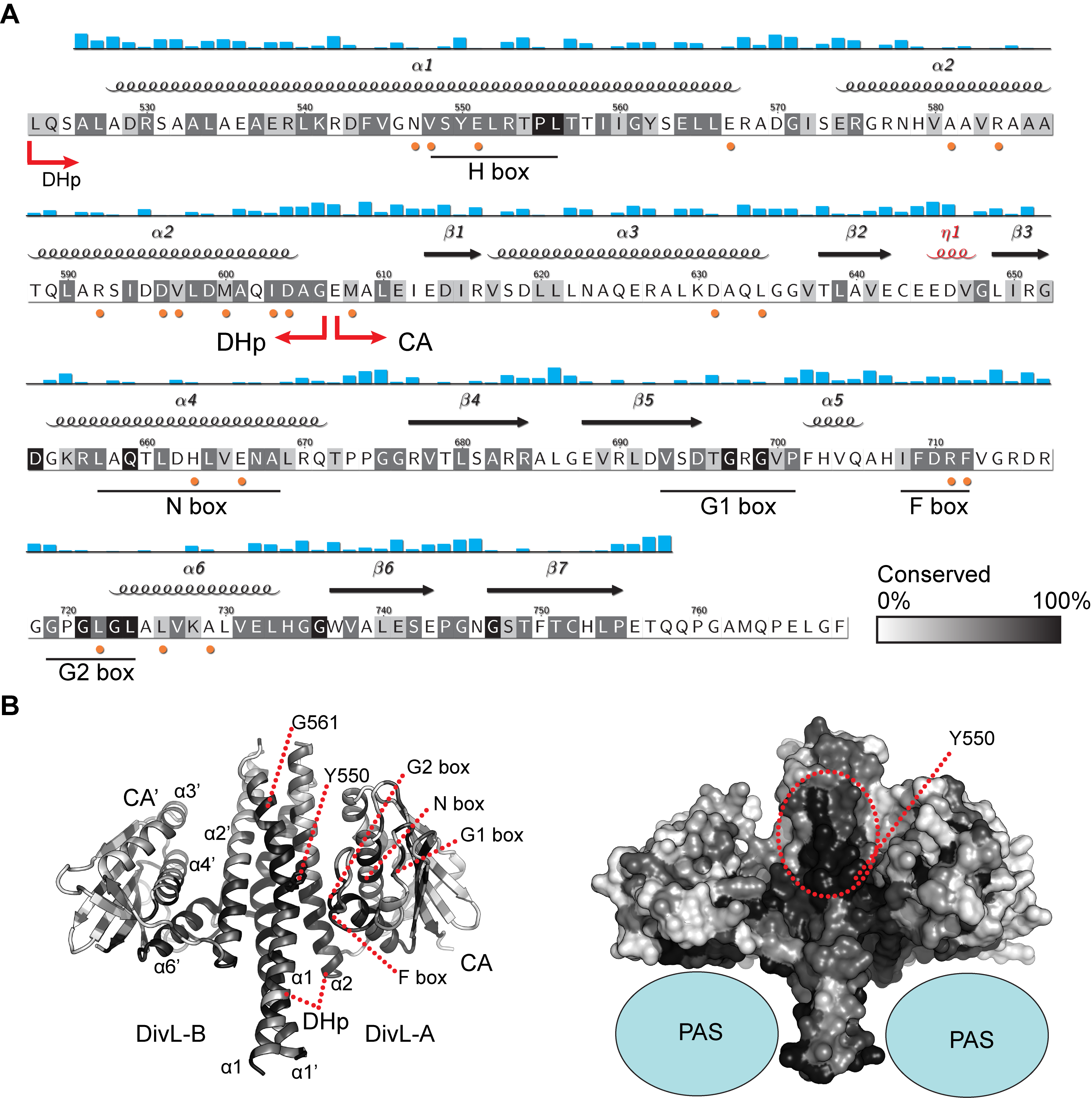

Supplement: Figure S2 — Sequence conservation of DivL orthologs mapped on the DivL structure. (A) The sequence of the HK region of DivL mapped with the secondary structures. The sequence is colored in gradient by degrees of sequence conservation, calculated from top 40 DivL homologs with sequence identity between 35% and 90%, from not conserved (white background) to strictly conserved (black background). The relative solvent accessibility of each residue (ratio of solvent accessible surfaces of each residue in the structure and in solution) is shown by blue boxes above the sequence. The residues that are near the DHp-CA interface in the structure shown in Figure S3 are marked by orange dots below the sequence. The five sequence motifs (H, N, G1, F, and G2) defined by Parkinson and Kofoid [67] are marked at the bottom. (B) Mapping of sequence conservation as a grayscale with black (most conserved), white (non-conserved) onto the DivL dimer, shown in cartoon (left) and surface (right) representations. (TIF) [file pbio.1001979.s002.tif]

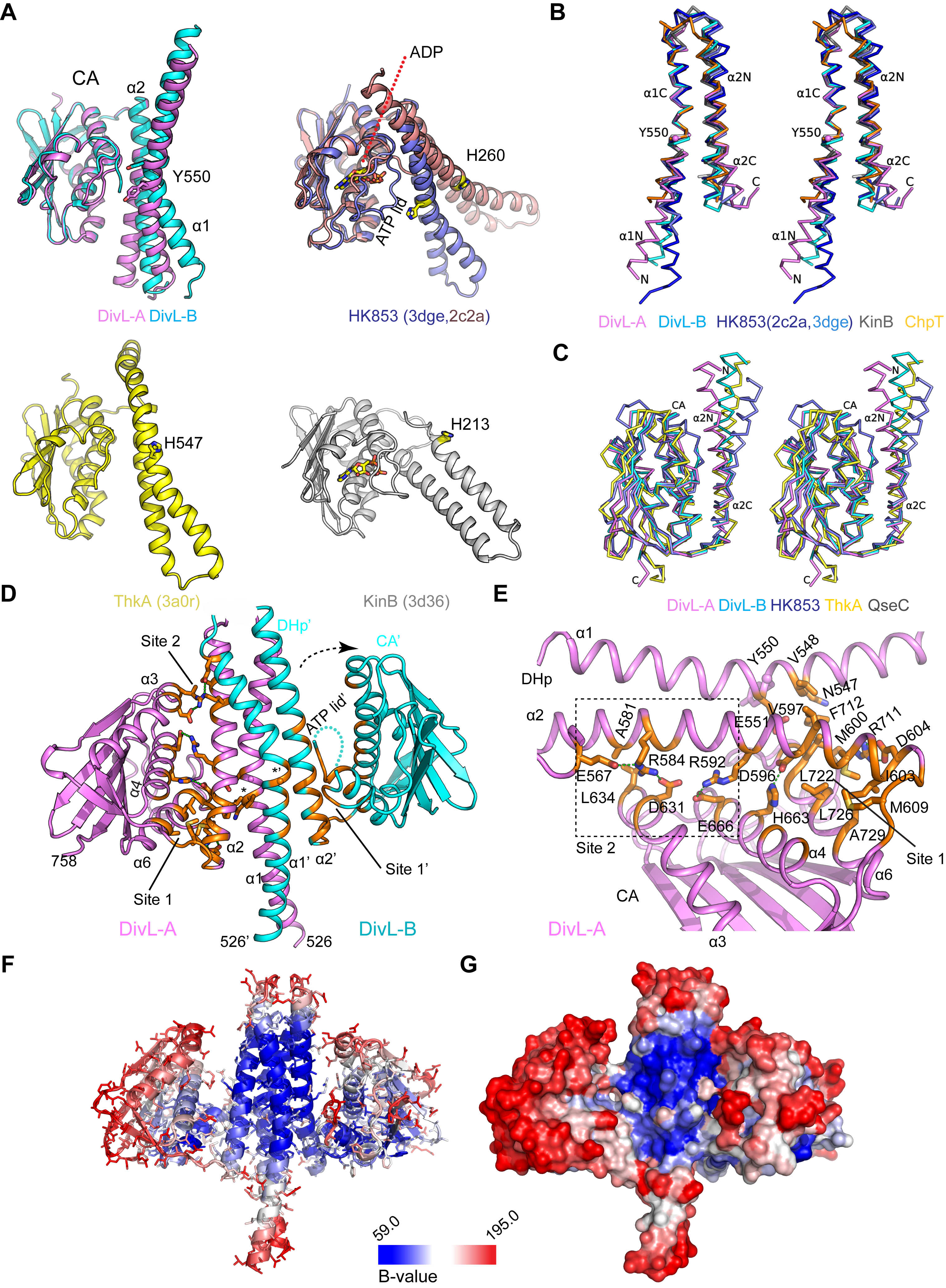

Supplement: Figure S3 — Structure comparisons, domain interface between DHp and CA, and B-value distribution of DivL. (A) Structural comparison of DivL to HK853 (phosphotransfer/phosphatase state, PDB code 3dge [23]), ThkA (PDB code 3a0r[25]), and KinB (PDB code 3d36 [31]). The highly conserved CA domains in all structures are shown in the same orientation. Residues at the phosphorylation site and ADP are shown in sticks. (B) Stereoview of the “RR docking modules” in HKs (HK853 and KinB) and ChpT. The structures were superposed using a stretch of 15 residues starting from the phosphorylation site. (C) Stereoview of the substructures that include 〈2C and the CA domain in DivL-A, DivL-B, HK853, QseC (PDB code 3jz3), and ThkA. (D) Overview of the DHp-CA interface in the DivL dimer. The residues near the domain interface are highlighted in orange. (E) A close-up view of the DHp-CA interactions in DivL-A. (F–G) Distribution of B-values in the DivL structure, shown in cartoon (F) and surface (G) representations. The B-values are colored in a gradient from low (blue) to high (red). (TIF) [file pbio.1001979.s003.tif]

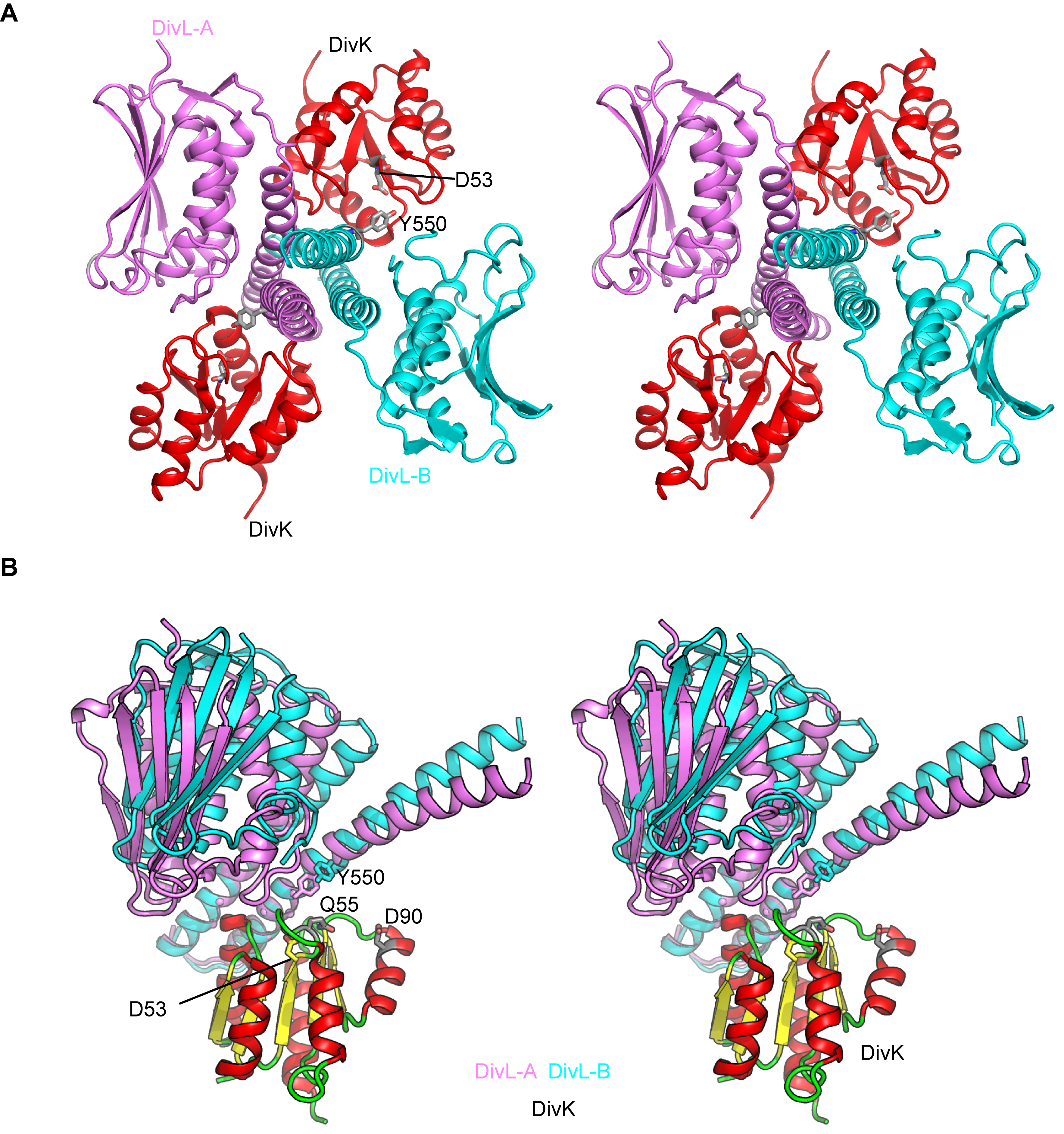

Supplement: Figure S4 — A model of DivL-DivK interaction. (A) A model of DivL and DivK interaction, shown in stereoview. The model is based on the HK853 complex with its cognate RD RR468 (PDB code 3dge). DivK (PDB code 1mav) is shown in red, and DivL in violet and cyan. The phosphorylatable residue Asp53 of DivK and Tyr550 of DivL are shown as sticks. (B) DivL-A/DivK (violet/red) and DivL-B/DivK (cyan/red) are superimposed using the “RR docking modules” and DivK, indicating that structural changes in DivL can impact its interactions with DivK. Asp53, Gln55, and Asp90 of DivK and Tyr550 of DivL are shown as sticks. (TIF) [file pbio.1001979.s004.tif]

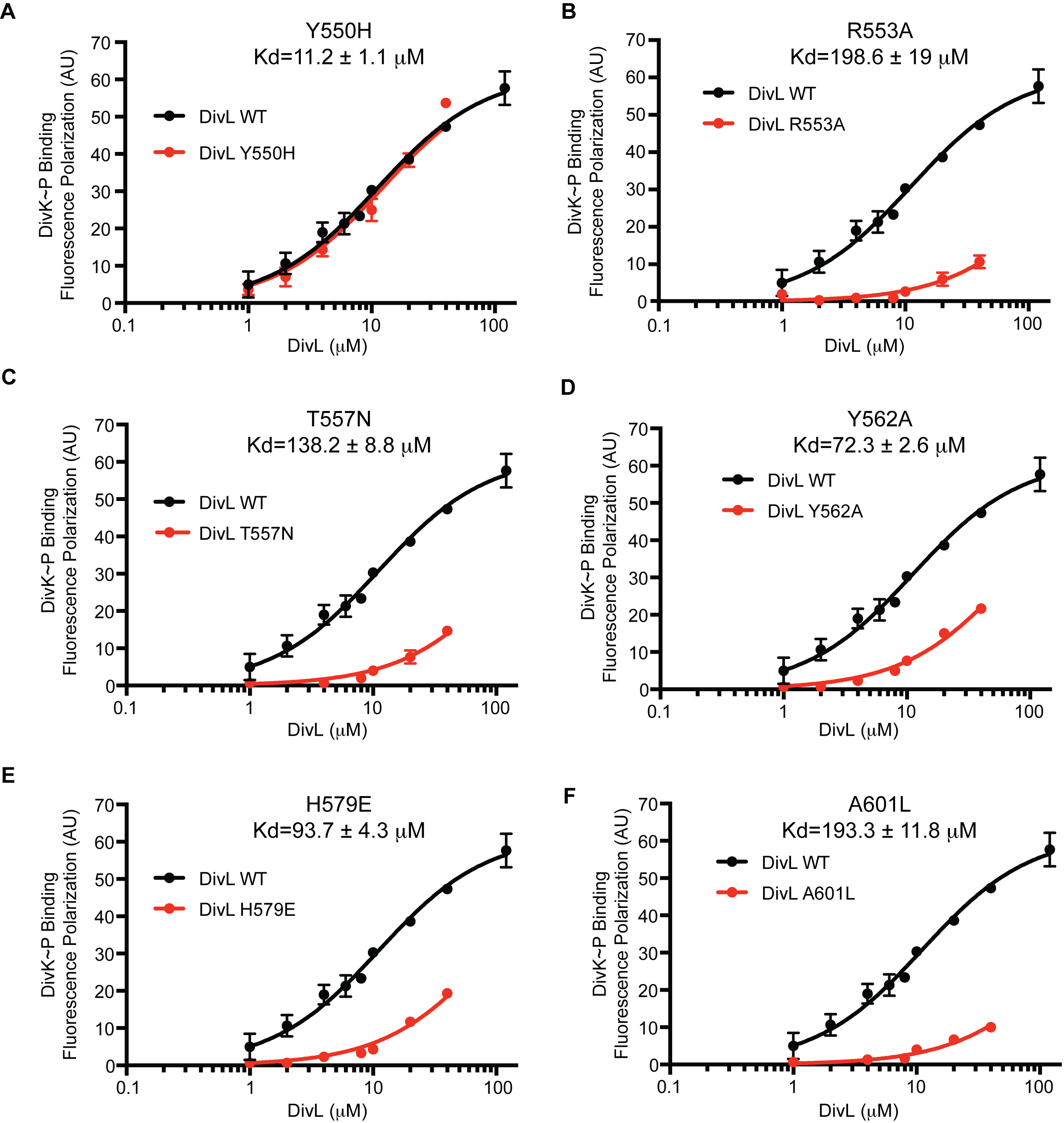

Supplement: Figure S5 — Binding curves for DivL(152–769) binding mutants to DivK∼P (red curves) and compared to wild-type DivL (black curves) for the following point mutations: (A) Y550H, (B) R553A, (C) T557N, (D) Y562A, (E) H579E, and (F) A601L. Numerical data used to generate manuscript graphs or histograms can be found in Table S1. (TIF) [file pbio.1001979.s005.tif]

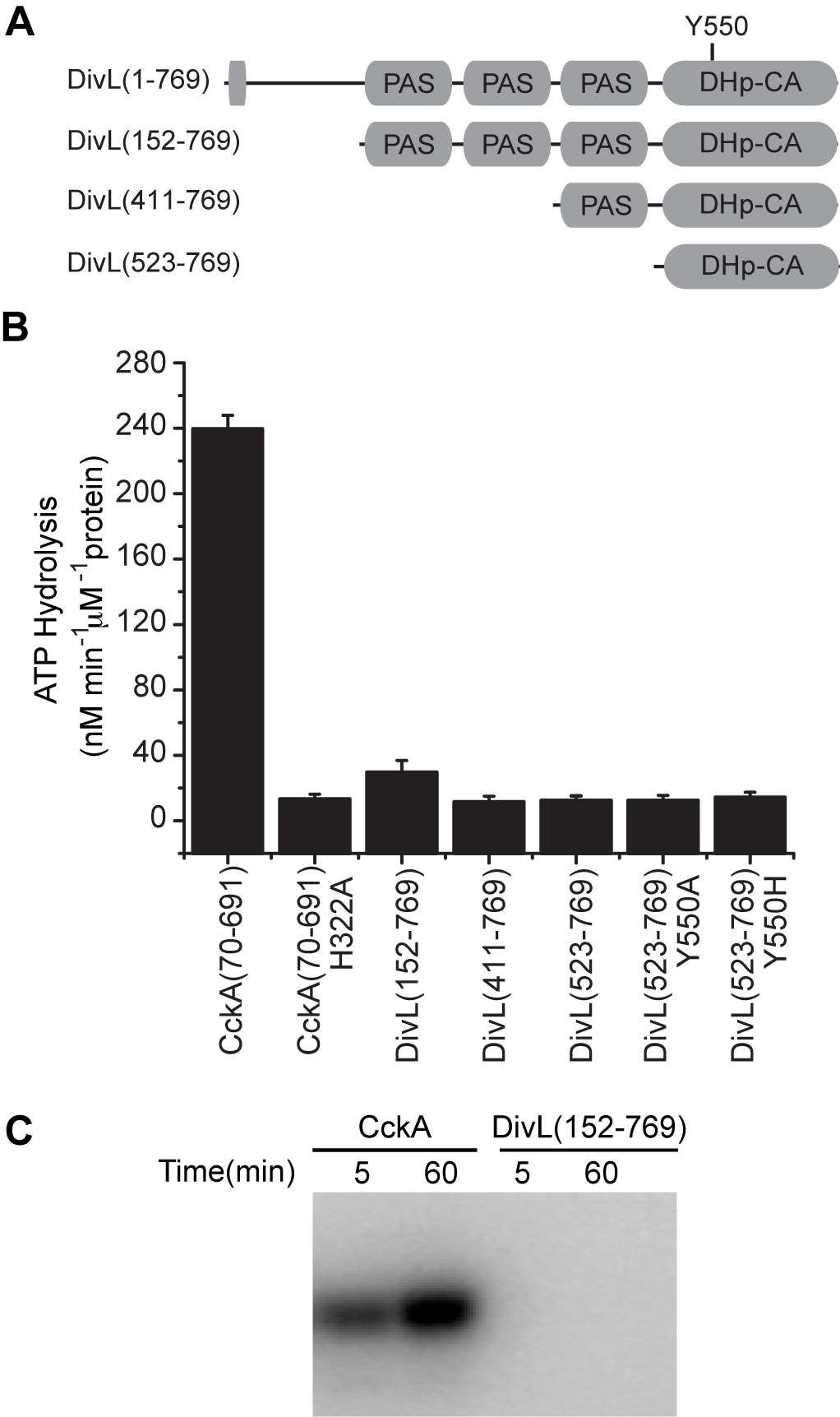

Supplement: Figure S6 — DivL has no kinase activity in vitro . (A) DivL constructs used in the assay. (B) Comparison of kinase activities of various DivL constructs relative to the CckA kinase controls. NADH coupled enzyme assay compares the NADH consumption rate at 5 µM concentration for each protein. (C) Comparison of autophosphorylation reactions of 5 µM CckA(70–691) or DivL(152–769) in kinase buffer supplemented with [γ-32P] ATP and 5 mM MgCl2. Numerical data used to generate manuscript graphs or histograms can be found in Table S1. (TIF) [file pbio.1001979.s006.tif]

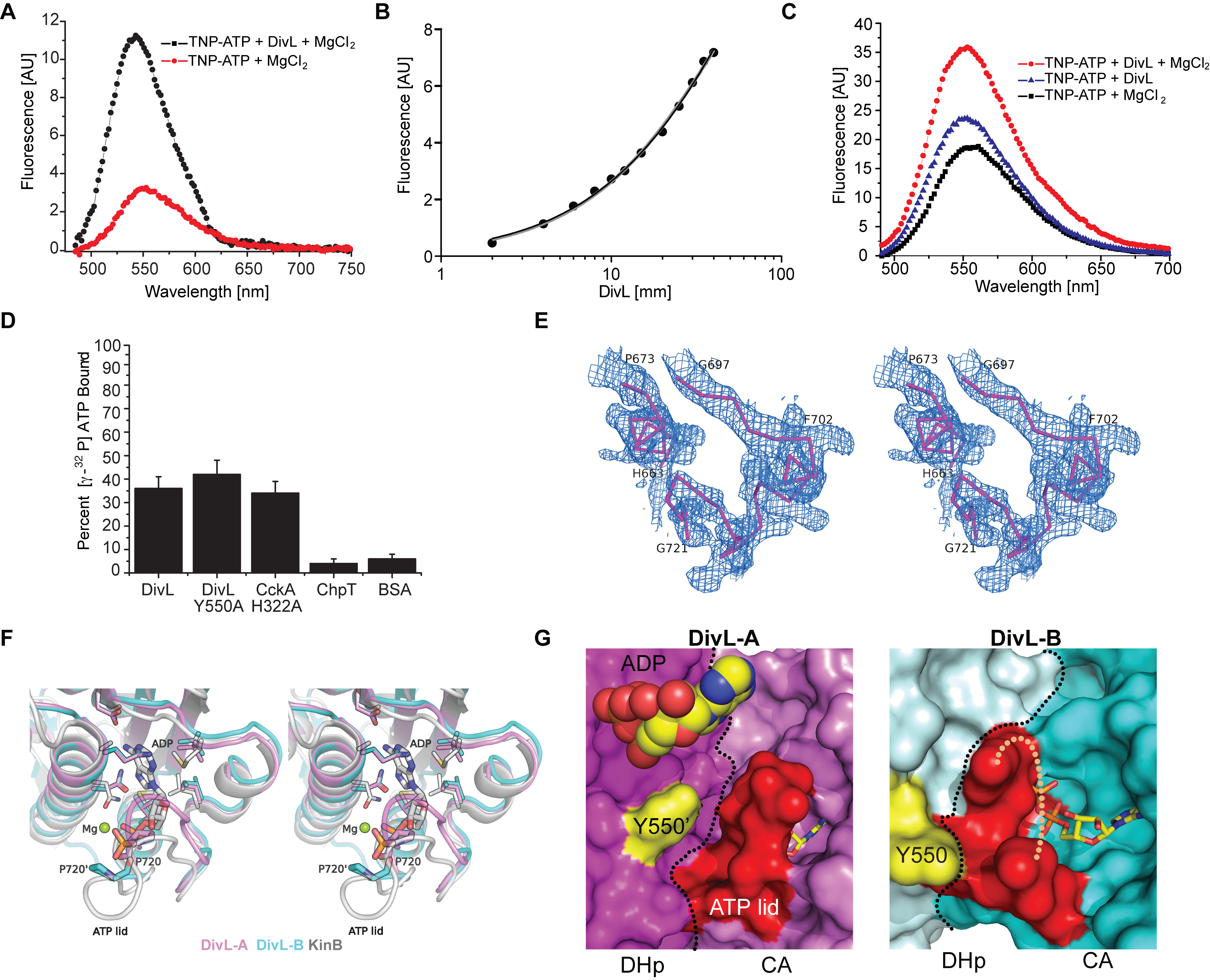

Supplement: Figure S7 — Nucleotide-binding assays. (A) Binding of 1 µM TNP-ATP (red) compared with 10 µM DivL and 1 µM TNP-ATP (black). (B) Titration of 2–40 µM DivL into 0.5 µM TNP-ATP for binding constant estimate of 57±8 µM, Bmax of 18, and R2 = 0.9976. (C) The impact of MgCl2 on the binding of TNP-ATP to DivL. Fluorescence emission profile of 5 µM TNP-ATP (black), 5 µM TNP-ATP+5 µM DivL (blue), 5 µM TNP-ATP+5 µM DivL+5 mM MgCl2 (red). (D) Filter binding assay indicating percent of 27.5 fmol of [γ-32P] ATP bound to 10 µM DivL(523–769), 10 µM DivL(523–769) Y550A, CckA(70–691) H322A, ChpT, and BSA. (F–G) ATP binding site in DivL. (E) Stereoview of the 2Fo-Fc electron density map near the nucleotide binding region of the ADP and Mg soaked crystal, contoured at 1.0 σ (blue). The refined model is shown as a Cα trace (magenta). (F) Stereoview of the ATP binding site of DivL-A (violet), DivL-B (cyan), and KinB (gray). All structures are superposed together using the CA domains. ADP and Mg molecules from KinB are shown. The residues near the ADP molecule are shown in sticks. (G) The ATP binding site on the DivL-A (left) and DivL-B (right). Each monomer is colored in violet and cyan. An ADP molecule, shown as sticks, was modeled into the ATP binding site based on the structure of KinB. The ATP lid is colored in red. Tyr550 is colored in yellow. An ADP molecule, shown in spheres in the same scale, highlights the size of ADP compared to the opening for ATP entry. Numerical data used to generate manuscript graphs or histograms can be found in Table S1. (TIF) [file pbio.1001979.s007.tif]

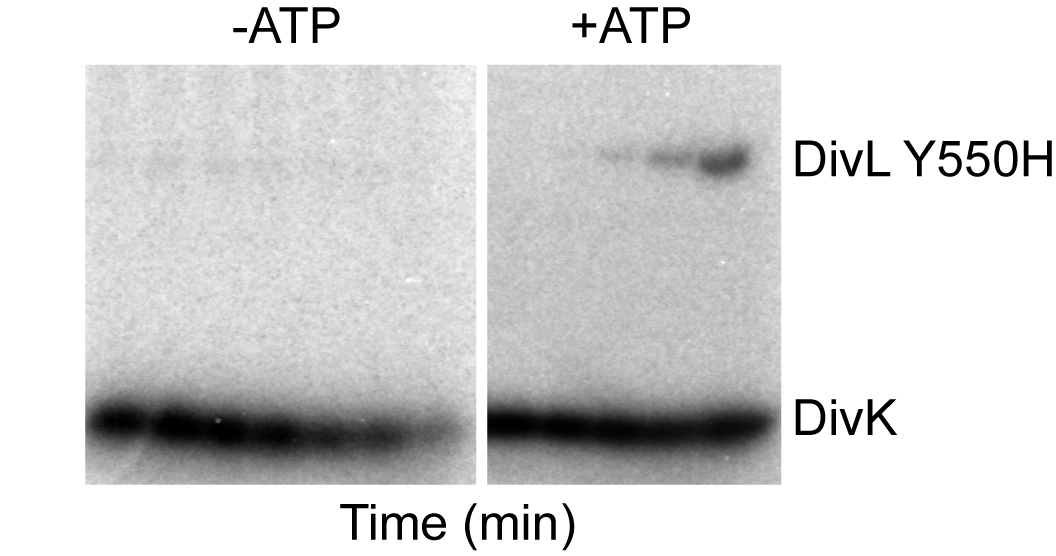

Supplement: Figure S8 — DivL(152–769) Y550H accumulates phosphate in a DivK∼P and ATP-dependent manner. Comparison of reaction mixtures of DivL(152–769) Y550H with phosphorylated DivK∼P in the presence and absence of ATP. (TIF) [file pbio.1001979.s008.tif]

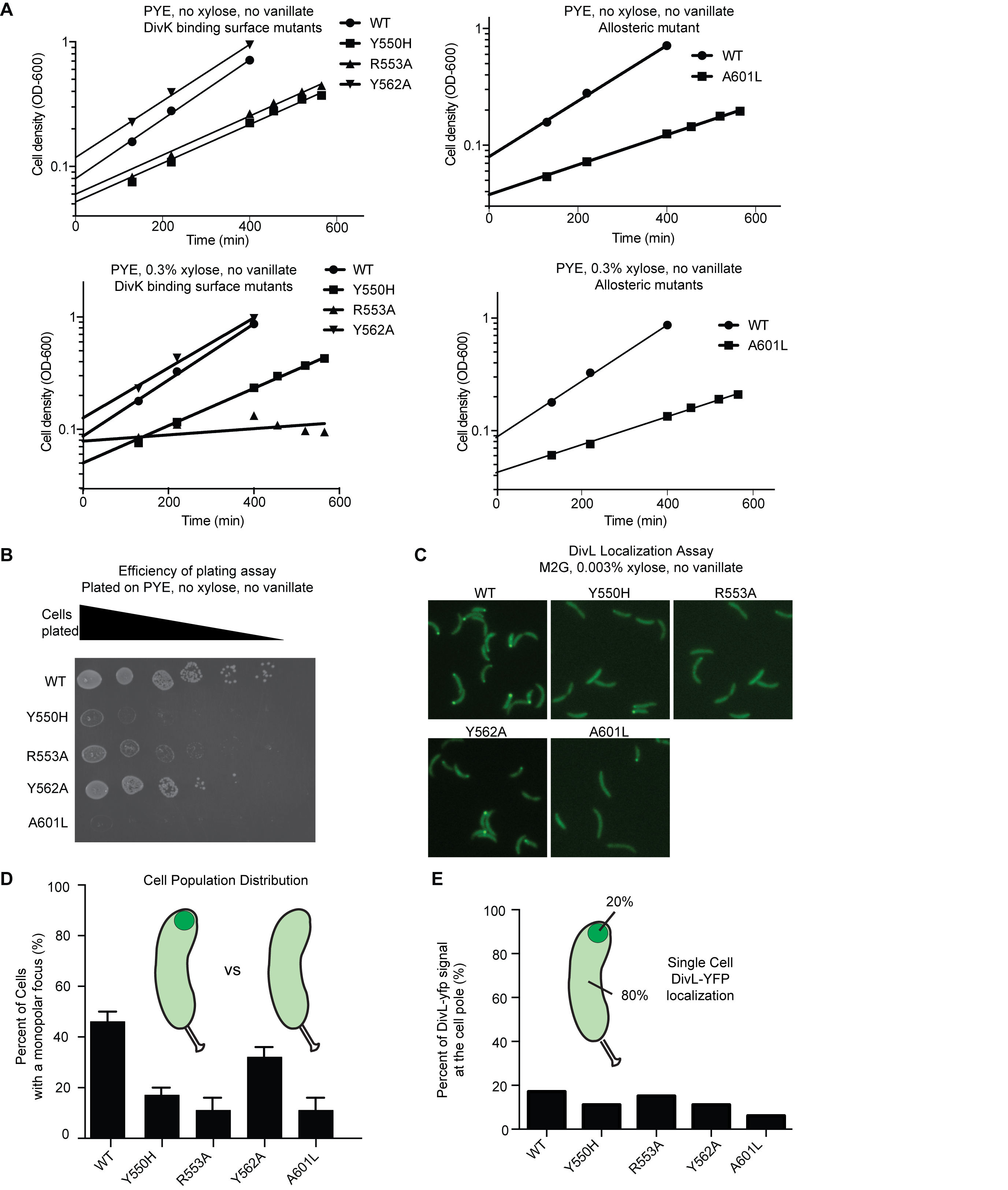

Supplement: Figure S9 — Effects on Caulobacter growth and DivL localization by site-directed mutations disrupting DivL-DivK interaction. (A) Growth curves of strains containing divL point mutants. Growth was measured by optical density for 10 hours or until culture saturation. Mutants were binned depending on their location on the RR binding surface or based on allostery involving the input-output helix of DivL. Growth curves were consistent across triplicate measurements, and one representative curve is shown for each mutant grown with and without xylose. Curves were fitted to a single exponential model. Inset: cartoon of DivL depicting the approximate location of each mutant. (B) Cell growth assayed by efficiency of plating. Prior to plating, cells were grown in PYE liquid media to OD-600 of 0.2 in the absence of xylose for 6 h. The culture was diluted 10-fold for the left-most inoculation, and 5-fold for subsequent dilutions. Plating was performed in duplicate using no xylose concentrations or vanillate. A representative plate is shown. (C–E) DivL-DivK binding mutants impact DivL-yfp subcellular localization. divL-yfp mutant strains were grown in M2G supplemented with 0.003% xylose. (C) Fluorescent images of divL-yfp mutant strains were grown in M2G supplemented with 0.003% xylose for DivL-DivK binding mutants: Y550H, R553A, Y562A, and A601L relative to wild-type divL-yfp. (D) Cell population analysis characterizing the percentage of cells with an observable monopolar focus. (E) Single-cell analysis of the fraction of DivL-yfp signal localized at the cell pole in cells with an observable monopolar focus. Numerical data used to generate manuscript graphs or histograms can be found in Table S1. (TIF) [file pbio.1001979.s009.tif]
