# Supplementary material for: Cell Fate Regulation Governed by a Repurposed Bacterial Histidine Kinase
Source: PLoS Biol. 2014 Oct 28;12(10):e1001979. doi: 10.1371/journal.pbio.1001979 (PMC4211667; doi:10.1371/journal.pbio.1001979)
Supplement: Table S2 — Data collection and refinement statistics. (DOCX) [file pbio.1001979.s011.docx]

**Table S2: Data collection and refinement statistics**

| **Data collection** | Native | Au derivative | | | |
| --- | --- | --- | --- | --- | --- |
| Wavelength (Å) | 0.9795 | 1.0436 | 1.0397 | 1.04 | 0.8731 |
| Resolution range (Å) | 44.1-2.5 | 44.1-3.4 | 44.1-3.2 | 44.1-3.21 | 44.1-3.3 |
| No. observations | 137,256 | 41,079 | 47,510 | 47,524 | 44,299 |
| No. unique reflections | 19,457 | 8,052 | 9,518 | 9,513 | 8,784 |
| Completeness (%) | 99.3 (99.3)^a^ | 99.5 (99.9) | 98.9 (95.7) | 99.1 (97.1) | 99.5 (99.7) |
| Mean I/σ (I) | 28.1 (2.3)^a^ | 19.2 (1.7) | 16.4 (2.0) | 16.5 (2.0) | 13.9 (1.9) |
| R_merge_ on I (%) | 3.1 (97)^a^ | 5.6 (87.4) | 5.0 (73.8) | 5.0 (75.6) | 6.4 (85.2) |
| R_meas_ on I (%) | 3.4 (104) | 7.2 (108) | 6.2 (93.2) | 6.3 (95.3) | 8.0 (106) |
| R_pim_ on I (%) | 1.3 (38.0) | 4.1 (62.5) | 3.7 (56.3) | 3.7 (57.5) | 4.7 (62.0) |
| High resolution shell | 2.64-2.50 | 3.58-3.4 | 3.38-3.2 | 3.38-3.21 | 3.48-3.3 |
| **Model and refinement statistics** | | | | | |
| No. reflections (total) | 19,452 |  | | | |
| No. reflections (test) | 965 |  | | | |
| Cutoff criteria | \|F\|>0 |  | | | |
| R_cryst_ | 0.202 |  | | | |
| R_free_ | 0.232 |  | | | |
| Restraints (RMS observed) |  |  | | | |
| Bond angle (°) | 1.06 |  | | | |
| Bond length (Å) | 0.010 |  | | | |
| Molprobity Scores |  |  | | | |
| All-atom clash score | 1.32 |  | | | |
| Ramachandran plot  Favored (allowed, %) | 97.5 (100) |  | | | |
| Rotamer outliers (%) | 1.2 |  | | | |
| Average isotropic B-value (Å^2^) | 107.1 (90.0)^b^ |  | | | |
| ESU based on R_free_ (Å) | 0.24 |  | | | |
| Protein residues / atoms | 460 / 3435 |  | | | |

^a^Highest resolution shell in parentheses. The high resolution cutoff was chosen such that the mean I/σ(I) in the highest resolution shell is around 2.

^b^This value represents the total B that includes TLS and residual B components. Wilson B-value in parenthesis.

R_merge_= Σ_hkl_ Σ_i_|I_i_(hkl)-<I(hkl)>|/ Σ_hkl_ Σ _i_I_i_(hkl), R_meas_(redundancy-independent R_merge_) = Σ_hkl_[N_hkl_/(N_hkl_-1)]^1/2^ Σ_i_|I_i_(hkl)-<I(hkl)>|/Σ_hkl_ Σ_i_I_i_(hkl), and R_pim_(precision-indicating R_merge_)= Σ_hkl_[1/(N_hkl_-1)]^1/2^ Σ_i_|I_i_(hkl)-<I(hkl)>|/Σ_hkl_ Σ_i_I_i_(hkl).

R_cryst_ = Σ| |F_obs_|-|F_calc_| | / Σ|F_obs_|, where F_calc_ and F_obs_ are the calculated and observed structure factor amplitudes, respectively. R_free_ = as for R_cryst_, but for 5.0% of the total reflections chosen at random and omitted from refinement.

ESU = Estimated overall coordinate error.
