# Supplementary material for: Cell Fate Regulation Governed by a Repurposed Bacterial Histidine Kinase
Source: PLoS Biol. 2014 Oct 28;12(10):e1001979. doi: 10.1371/journal.pbio.1001979 (PMC4211667; doi:10.1371/journal.pbio.1001979)
Supplement: Table S3 — DNA oligos used in this study. (DOCX) [file pbio.1001979.s012.docx]

**Table S3. DNA oligos used in this study**

| Name | Description | Site | Sequence 5’-3’ (endonuclease site or mutation site underlined) |
| --- | --- | --- | --- |
| T7 |  |  | TAATACGACTCACTATAGG |
| T7term |  |  | GCTAGTTATTGCTCAGCG |
| WSCp8 |  | EcoR1 | AAAA**GAATTC**CTAGAAGCCGAGTTCGGGCTGCA |
| WSCp10 |  | NdeI | AAAA**CATATG**CTGGACAAGCTGAACGACGC |
| WSCp11 |  | NdeI | AAAA**CATATG**CTGCAGAGCGCCCTGGCCGAT |
| WSCp31 |  | NdeI | AAAACATATGCGCGCCGACTCGGGCCT |
| WSCp34 | His-DivL(152-769) |  | T**GAATTC**CTAGAAGCCGAGTTCGGGCTGCATGGCC |
| WSCp35 | His-DivL(281-769) | NdeI | GAATCAG**CATATG**ACCCTGAACCACATCGCCGAGGCC |
| WSCp36 | His-DivL(54-769) | NdeI | GAATCAG**CATATG**GCCTGGCTCGACGCCTTCGACA |
| WSCp39 | His-DivK |  | CA**GCTAGC**ATGGTCGGGGTAAGGATGACGAAGAA |
| WSCp40 | His-DivK |  | TT**GAGCTC**TCATGCAGGCTGCCTTTCCAG |
| WSCp43 |  | Y550A | CGGCAATGTCTCC**GCC**GAGCTGCGCACGC |
| WSCp44 |  | Y550A | GCGTGCGCAGCTC**GGC**GGAGACATTGCCG |
| WSCp45 |  | Y550H | GCGTGCGCAGCTC**GTG**GGAGACATTGCCG |
| WSCp46 |  | Y550H | CGGCAATGTCTCC**CAC**GAGCTGCGCACGC |
| WSCp65 |  | Y562A | CAGCAGCTCCGA**GGC**GCCGATGATCGT |
| WSCp73 |  | Y562A | ACGATCATCGGC**GCC**TCGGAGCTGCTG |
| WSCp66 |  | R553A | CGTCAGCGGCGT**GGC**CAGCTCGTAGGA |
| WSCp74 |  | R553A | TCCTACGAGCTG**GCC**ACGCCGCTGACG |
| WSCp67 |  | A601L | CGGCGTCGATCTG**CAG**CATGTCCAGCACG |
| WSCp75 |  | A601L | CGTGCTGGACATG**CTG**CAGATCGACGCCG |
| WSCp112 | His-DivJ(195-596) | NheI | AAAGCTAGCCGCGACGATCGCTACGCCAG |
| WSCp113 | His-DivJ(195-596) | SacI | AAAGAGCTCCCTCAGCGCGGCGCAAAGG |
| WSCp114 | His-PleC(310-842) | NheI | AAAAGCTAGCGTCGCCCATCGCGAGTTCATCG |
| WSCp115 | His-PleC(310-842) | SacI | AAAAGAGCTCCCTCAGGCCGCCACGAAGTC |
| WSCp199 |  | T557N | CTGCGCACGCCGCTGAACACGATCATCGGCTATTC |
| WSCp200 |  | T557N | GAATAGCCGATGATCGTGTTCAGCGGCGTGCGCAG |
| WSCp197 |  | H579E | GAGCGGGGCCGCAACGAAGTGGCCGCCGTCCG |
| WSCp198 |  | H579E | CGGACGGCGGCCACTTCGTTGCGGCCCCGCTC |
| WSCp10024 |  |  | CCTTAAGATCTCGAGCTCCGGAGAATATGACTTCGTACGACCTGATCC |
| WSCp10025 |  |  | GCCGACCGGTGACGCGTAACGTTCGAGAAGCCGAGTTCGGGCTGC |
| WSCp10062 |  |  | CTAGTGAAAACCTGTATTTTCAGGGCCGCGCCGACTCGGGCCTG |
| WSCp10063 |  |  | GAGAATTCCATGGCCATATGGCTAGCCTAGAAGCCGAGTTCGGGC |
| WSCp10022 |  |  | ACCACGTCAGAGAATCTCTACTTCCAAGGATGACGAAGAAGGTCCTCATCGTGG |
| WSCp10023 |  |  | ACCACGTCAGAGAATCTCTACTTCCAAGGATGACGAAGAAGGTCCTCATCGTGG |
| JABp64 |  | NdeI | GGCCTTGTCCATATGCGCGGCTCAGCGCTTTCCGG |
| JABp66 |  | SacI | ACTGCGGAGCTCCTACGCCGCCTGCAGCTGCTG |
| JABp68 |  | NdeI | CCAAG**CATATG**CGTTATTTTCTCCAAGCGC |
| JABp69 |  | SacI | T**GAGCTC**TTACGCCGGGACCCAG |
| JABp72 |  | NdeI | CGCC**CATATG**ACCGAGACCGTC |
| JABp73 |  | NcoI | GCC**CCATGGG**CGGCTCAGCGCTTTCCGGC |
| JABp74 |  | SacI | GC**GAGCTC**GCCGCCTGCAGCTGCTGCTTGAC |
| JABp85 |  |  | CAGCCGGATCTCAGTG |
| JABp86 |  |  | TCGAGATCTCGATCCCGCGAAATTAATACGACTCACT |
| JABp88 |  | H322A | TCGCG**GCC**GACTTCAA |
