# Supplementary material for: Cell Fate Regulation Governed by a Repurposed Bacterial Histidine Kinase
Source: PLoS Biol. 2014 Oct 28;12(10):e1001979. doi: 10.1371/journal.pbio.1001979 (PMC4211667; doi:10.1371/journal.pbio.1001979)
Supplement: Table S4 — Plasmids and strains used in this study. (DOCX) [file pbio.1001979.s013.docx]

**Table S4. Plasmids and strains used in this study**

| Plasmid | Description | Reference |
| --- | --- | --- |
| pET-28b(+) | bacterial expression vector | Novagen |
| pTEV5 | bacterial expression vector | ^13^ |
| pTEV6 | bacterial expression vector with MBP solubilization tag | (17) |
| pJAB27 | pET-28b(+) CckA(70-691) expression vector | This Study |
| pJAB30 | pET-28b(+) ChpT expression vector | This Study |
| pJAB35 | pET-28b(+) CckA(70-691) H322A expression vector | This Study |
| pWSC7 | pET-28b(+) DivL(411-769) expression vector | This Study |
| pWSC8 | pET-28b(+) DivL(523-769) expression vector | This Study |
| pWSC24 | pET-28b(+) DivL(152-769) expression vector | This Study |
| pWSC25 | pET-28b(+) DivL(281-769) expression vector | This Study |
| pWSC26 | pET-28b(+) DivL(54-769) expression vector | This Study |
| pWSC27 | pET-28b(+) DivL(523-769) A601L expression vector | This Study |
| pWSC28 | pET-28b(+) DivL(523-769) R553A expression vector | This Study |
| pWSC29 | pET-28b(+) DivJ(195-596) | This Study |
| pWSC30 | pET-28b(+) PleC(310-842) | This Study |
| pWSC31 | pET-28b(+) DivK | This Study |
| pWSC43 | pET-28b(+) DivL(523-769) Y550H expression vector | This Study |
| pWSC10036 | pTEV5-DivL(152-769) | This Study |
| pWSC10040 | pTEV5-DivL(152-769) Y550H | This Study |
| pWSC10046 | pTEV5-DivL(152-769) R553A | This Study |
| pWSC10048 | pTEV5-DivL(152-769) T557N | This Study |
| pWSC10041 | pTEV5-DivL(152-769) Y562A | This Study |
| pWSC10045 | pTEV5-DivL(152-769) H579E | This Study |
| pWSC10038 | pTEV5-DivL(152-769) A601L | This Study |
| pWSC10039 | pTEV6-DivK | This Study |
| pWSC10010 | pXYFPC1_DivL | This Study |
| pWSC10023 | pXYFPC1_DivL Y550H | This Study |
| pWSC10027 | pXYFPC1_DivL R553A | This Study |
| pWSC10026 | pXYFPC1_DivL Y562A | This Study |
| pWSC10028 | pXYFPC1_DivL A601L | This Study |
| *C. crescentus* NA1000 | Laboratory Caulobacter crescentus strain | (18) |
| *E. coli* DH5α | Bacterial cloning strain | Invitrogen |
| E. coli  Rosetta(DE3)pLysS | Bacterial expression strain | Novagen |
| WSC226 | pET-28b(+) DivJ expression vector | This Study |
| WSC229 | pET-28b(+) PleC expression vector | This Study |
| WSC224 | pET-28b(+) DivK expression vector | This Study |
| AA871 | ΔdivL vanA::divL | (19) |
| WSC399 | ΔdivL vanA::divL xylX::divL-yfp | This Study |
| WSC308 | ΔdivL vanA::divL xylX::divL-yfp Y550H | This Study |
| WSC310 | ΔdivL vanA::divL xylX::divL-yfp R553A | This Study |
| WSC312 | ΔdivL vanA::divL xylX::divL-yfp Y562A | This Study |
| WSC314 | ΔdivL vanA::divL xylX::divL-yfp A601L | This Study |
| LS101 | Caulobacter crescentus NA1000 | (20) |
| LS4468 | Caulobacter CB15N divL::divL-m2 | (19) |
