# Supplementary material for: Cell Fate Regulation Governed by a Repurposed Bacterial Histidine Kinase
Source: PLoS Biol. 2014 Oct 28;12(10):e1001979. doi: 10.1371/journal.pbio.1001979 (PMC4211667; doi:10.1371/journal.pbio.1001979)
Supplement: Table S5 — Gibson cloning strategy to generate His-DivL(152–769) point mutants into pTEV5 for protein expression. (DOCX) [file pbio.1001979.s014.docx]

**Table S5. Gibson cloning strategy to generate His-DivL(152-769) point mutants into pTEV5 for protein expression**

| Plasmid # | DivL Mutation | First Fragment Codons | Primer 1 | Primer 2 | Second  Fragment Codons | Primer 1 | Primer 2 |
| --- | --- | --- | --- | --- | --- | --- | --- |
| pWSC10040 | Y550H | 152-554 | WSCp10062 | WSCp62 | 546-769 | WSCp70 | WSCp10063 |
| pWSC10046 | R553A | 152-557 | WSCp10062 | WSCp66 | 549-769 | WSCp74 | WSCp10063 |
| pWSC10048 | T557N | 152-563 | WSCp10062 | WSCp200 | 552-769 | WSCp199 | WSCp10063 |
| pWSC10041 | Y562A | 152-566 | WSCp10062 | WSCp65 | 558-769 | WSCp73 | WSCp10063 |
| pWSC10045 | H579E | 152-584 | WSCp10062 | WSCp198 | 574-584 | WSCp197 | WSCp10063 |
| pWSC10038 | A601L | 152-606 | WSCp10062 | WSCp67 | 597-769 | WSCp75 | WSCp10063 |
