# Supplementary material for: Cell Fate Regulation Governed by a Repurposed Bacterial Histidine Kinase
Source: PLoS Biol. 2014 Oct 28;12(10):e1001979. doi: 10.1371/journal.pbio.1001979 (PMC4211667; doi:10.1371/journal.pbio.1001979)
Supplement: Table S6 — Gibson cloning strategy to generate DivL-yfp point mutants into the pXYFPC-1 vector that is designed to integrate at the xylose chromosomal locus using the Gibson DNA assembly method. (DOCX) [file pbio.1001979.s015.docx]

**Table S6.** Gibson cloning strategy to generate DivL-yfp point mutants into the pXYFPC-1 vector that is designed to integrate at the xylose chromosomal locus using the Gibson DNA assembly method.

| Plasmid # | DivL Mutation | First Fragment Codons | Primer 1 | Primer 2 | Second  Fragment Codons | Primer 1 | Primer 2 |
| --- | --- | --- | --- | --- | --- | --- | --- |
| pWSC10023 | Y550H | 152-554 | WSCp10024 | WSCp62 | 546-769 | WSCp70 | WSCp10025 |
| pWSC10027 | R553A | 152-557 | WSCp10024 | WSCp66 | 549-769 | WSCp74 | WSCp10025 |
| pWSC10026 | Y562A | 152-566 | WSCp10024 | WSCp65 | 558-769 | WSCp73 | WSCp10025 |
| pWSC10028 | A601L | 152-606 | WSCp10024 | WSCp67 | 597-769 | WSCp75 | WSCp10025 |
