# Supplementary material for: Cell Fate Regulation Governed by a Repurposed Bacterial Histidine Kinase
Source: PLoS Biol. 2014 Oct 28;12(10):e1001979. doi: 10.1371/journal.pbio.1001979 (PMC4211667; doi:10.1371/journal.pbio.1001979)
Supplement: Table S7 — Accession numbers for genes and proteins. (DOCX) [file pbio.1001979.s016.docx]

**Table S7. Accession Numbers for Genes and Proteins.**

| Protein Name | Caulobacter crescentus NA1000 Gene Number* | SwissProt Accession Number | Pubmed Accession Number |
| --- | --- | --- | --- |
| DivL | CCNA_03598 (CC_3484) | B8H5L8 | ACL97063 |
| DivK | CCNA_02547 (CC_2463) | B8GZM3 | ACL96012 |
| CckA | CCNA_01132 (CC_1078) | B8H3H8 | ACL94597 |
| ChpT | CCNA_03584 (CC_3470) | B8H5K4 | ACL97049 |
| CtrA | CCNA_03130 (CC_3035) | B8H358 | ACL96595 |
| DivJ | CCNA_01116 (CC_1063) | B8H3G2 | ACL94581 |
| PleC | CCNA_02567 (CC_2482) | B8H024 | ACL96032 |

* *Caulobacter crescentus* strains CB15 gene number shown in parenthesis.
