# Supplementary material for: Cell Fate Regulation Governed by a Repurposed Bacterial Histidine Kinase
Source: PLoS Biol. 2014 Oct 28;12(10):e1001979. doi: 10.1371/journal.pbio.1001979 (PMC4211667; doi:10.1371/journal.pbio.1001979)
Supplement: Text S1 — Supplementary discussion of the DivL structure. (DOCX) [file pbio.1001979.s017.docx]

**Cell Fate Regulation Governed by a Repurposed Bacterial Histidine Kinase**

**Text S1**

***Bacterial pseudo HKs*** Pseudo histidine kinases are expected to retain partial HK function, for example, as a phosphotransferase or a phosphatase. Some phosphotransferases, such as Spo0B and ChpT [1,2], adopt an HK-like structure that retains the phosphorylatable histidine but no longer possesses the ATP binding site. Another less divergent group of easily identifiable pseudo HKs consists of HKs without the phosphorylatable histidine in the H-box. This subset of pseudo HKs account for ~1.2 % of the total HK population. Within the pseudo-HK sub-population, ~49% of are found in bacteria, while 49% are in eukaryotes, and the remaining ~2% in archaea. About 25% of all pseudo-HKs contain a tyrosine at the invariant His position, as is the case with DivL. Within α-proteobacteria pseudo HKs, the tyrosine substitution occurs more frequently (~89%), many of which are putative DivL-like pseudo-HKs.

***Structural comparisons to other HKs*** The HK region of DivL shares significant sequence similarities (sequence identity ~25%) to four other structurally characterized HKs, HK853 [3], KinB [4], QseC [5], and PAS-linked HK ThkA [6]. Most of these structures contain both the DHp and CA domains, while the DHp domain of QseC is largely disordered except for the α2C helix. The HK853 structure was determined in two states, by itself and co-crystallized as a complex with its cognate response regulator (phosphotransfer/phosphatase state). Structural comparisons reveal that the CA domain and DHp arrangements vary significantly among characterized HKs (Fig. S3a). The underlying motion linking the two conformation states of DivL is different from that of HK853. The inter-domain movement of HK853 mostly involves the hinge between the CA domain and the DHp domain [3], while the inter-domain movement of DivL revolves around the “kinase” active site. Overall, each monomer of DivL is most similar to HK853 in the phosphotransfer state (Fig. S3a, PDB code 3dge).

The structures of the α1C and α2N portions of the DHp domain (i.e. the RR docking module) are highly conserved in DivL, KinB, HK853, and the phosphotransfer protein ChpT, while larger structural differences are observed in α1N and α2C (Fig. S3b). A highly conserved CA domain and a very similar arrangement between α2C and CA can be identified in DivL, the HK853 phosphotransfer state, ThkA, and QseC (Fig. S3c), suggesting this structural arrangement is a conserved feature of HKs. In contrast, helical interactions between the α2C helix and the CA domain within KinB, the HK853 resting state [7] and ChpT deviate more significantly. The helical interactions between the DHp and the CA domain are likely important for HK function by coordinating the arrangement of the ATP lid, ATP binding site, and H-box. Mutational studies of HK853 on its DHp-CA interface significantly impacted its autokinase activity [7], suggesting this interface plays a role in both autokinase and phosphotransfer activities.

***CA and DHp interface*** The CA and DHp domain interfaces are formed by extensive interactions between highly conserved residues (Fig. S3, highlighted in orange), involving the packing of the helical face (α3, α4 and α6) and the ATP lid of the CA domain against the DHp domain (Fig. S3d-e). The interaction interface can be divided into two patches (Sites 1 and 2) based on the nature of interaction, sequence conservation, and spatial location. The primary site, conserved in both DivL-A and DivL-B, consists of mainly hydrophobic clusters from C-terminal portion of α2 (α2C) and α6 (Fig. S3d-e, Site 1). The linker region between α2 and the CA domain contains a methionine (Met609) that points to the hydrophobic cluster. Site 1 also includes three hydrogen bonds formed by two side-chain pairs Asp596 and His663, Asp604 and Arg711. A weaker secondary interface, which is present only in DivL-A involves mainly polar interactions between the N-terminal portion of α2 and C-terminal portions of α3 and α4 (Fig. S3d-e, Site 2) that appear less conserved. These differences in the DHp-CA interactions between DivL-A and B are a result of rigid body movement of the “α2C+CA” module away from the “RR docking module”.

**Supplemental References**

1. Blair JA, Xu Q, Childers WS, Mathews, II, Kern JW, et al. (2013) Branched signal wiring of an essential bacterial cell-cycle phosphotransfer protein. Structure 21: 1590-1601.

2. Zapf J, Sen U, Madhusudan, Hoch JA, Varughese KI (2000) A transient interaction between two phosphorelay proteins trapped in a crystal lattice reveals the mechanism of molecular recognition and phosphotransfer in signal transduction. Structure 8: 851-862.

3. Casino P, Rubio V, Marina A (2009) Structural insight into partner specificity and phosphoryl transfer in two-component signal transduction. Cell 139: 325-336.

4. Bick MJ, Lamour V, Rajashankar KR, Gordiyenko Y, Robinson CV, et al. (2009) How to switch off a histidine kinase: crystal structure of Geobacillus stearothermophilus KinB with the inhibitor Sda. J Mol Biol 386: 163-177.

5. Xie W, Dickson C, Kwiatkowski W, Choe S (2010) Structure of the cytoplasmic segment of histidine kinase receptor QseC, a key player in bacterial virulence. Protein Pept Lett 17: 1383-1391.

6. Yamada S, Sugimoto H, Kobayashi M, Ohno A, Nakamura H, et al. (2009) Structure of PAS-Linked Histidine Kinase and the Response Regulator Complex. Structure 17: 1333-1344.

7. Marina A, Waldburger CD, Hendrickson WA (2005) Structure of the entire cytoplasmic portion of a sensor histidine-kinase protein. Embo J 24: 4247-4259.
