# Supplementary material for: Cell Fate Regulation Governed by a Repurposed Bacterial Histidine Kinase
Source: PLoS Biol. 2014 Oct 28;12(10):e1001979. doi: 10.1371/journal.pbio.1001979 (PMC4211667; doi:10.1371/journal.pbio.1001979)
Supplement: Text S2 — Detailed methods for Construction of plasmids and strains. (DOCX) [file pbio.1001979.s018.docx]

**Text S2**

***DivL expression vectors*** Primers and plasmids used in this study are shown in Supplementary Tables S2-S3. A fragment of *divL* encoding residues 523-769 (used in crystallization) was amplified by PCR from the template cosmid 2G1 [1] using primers WSCp8 and WSCp11. These primers introduced NdeI and EcoR1 restriction sites at the 5’- and 3’-ends. This PCR fragment was digested with NdeI and EcoR1 and ligated into a similarly digested pET-28b(+) vector, resulting in plasmid pWSC8 that encodes an N-terminal His_6_-tag (MGSSHHHHHHSSGLVPRGSHM) fused to DivL(523-769). For simplicity, this construct will be referred to as DivL(523-769) or DivL throughout the main text.

Other DivL plasmids containing 4 to 1 PAS domains respectively were constructed in the same manner: 4xPAS-DHp-CA DivL(54-769) plasmid pWSC26 (primers WSCp36 and WSCp8), 3xPAS-DHp-CA DivL(152-769) plasmid pWSC24 (primers WSCp31 and WSCp8), 2xPAS-DHp-CA DivL(281-769) plasmid pWSC25 (primers WSCp35 and WSCp8), and 1xPAS-DHp-CA DivL(411-769) plasmid pWSC7 (primers WSCp10 and WSCp8). The sequences of the resulting plasmids were confirmed by primer extension sequencing using oligos T7 and T7term.

***DivL(523-769) Y550H, R553A, A601L expression vectors*** Sequenced overlap extension (SOE PCR) method [2] was used to generate Y550H, R553A, and A601L point mutations into DivL(523-769). For the DivL(523-769) Y550H mutation, the 5’ terminus of *divL* was amplified by PCR using plasmid cosmid 2G1 as a template and oligos WSCp45 and WSCp11 (5’ SOE PCR). The 3’ terminus of *divL* was similarly amplified with oligos WSCp8 and WSCp46 (3’ SOE PCR). These two first-round SOE PCR products were mixed 1:1 as a template for the second round SOE PCR, then amplified using oligos WSCp8 and WSCp11. The second-round SOE PCR product was digested with NdeI and EcoRI and ligated with similarly digested pET-28b(+) vector backbone, resulting in plasmid pWSC43. R553A plasmid pWSC27 (5’ SOE PCR primers: WSCp66 and WSCp11; 3' SOE PCR primers: WSCp8 and WSCp74) and A601A plasmid pWSC28 (5’ SOE PCR primers: WSCp67 and WSCp11; 3' SOE PCR primers: WSCp8 and WSCp75) were constructed in the same manner.

***DivJ expression vector*** A fragment of *divJ* encoding a DHp-CA DivJ construct, DivL(195-596), was amplified by PCR using genomic DNA as a template and primers WSCp112 and WSCp113 that introduced NheI and SacI restriction sites at the 5’- and 3’-ends. This PCR fragment was digested with NheI and SacI and ligated into similar digested pET-28b(+) vector, resulting in plasmid pWSC29 that encodes an N-terminal His_6_-tag (MGSSHHHHHHSSGLVPRGSHM) fused to DivJ(195-596).

***PleC expression vector*** A fragment of *pleC* encoding a PAS-DHp-CA DivJ construct, PleC(310-842), was amplified by PCR using genomic DNA as a template and primers WSCp114 and WSCp115 that introduced NheI and SacI restriction sites at the 5’- and 3’-ends. This PCR fragment was digested with NheI and SacI and ligated into similar digested pET-28b(+) vector, resulting in plasmid pWSC30 that encodes an N-terminal hexahistidine tag (MGSSHHHHHHSSGLVPRGSHM) fused to PleC(310-842).

***DivK expression vector*** His-MBP-DivK was generated in pTEV6 using the Gibson DNA assembly method [3]. Primers for Gibson reactions were designed using J5 DNA assembly design automation software [4,5]. The resulting plasmid encodes a hexahistidine tag, followed by maltose binding protein (MBP) and a TEV protease cleavage site at the N-terminus of DivK. Full length *divK* was amplified by PCR using genomic DNA as a template with primers WSCp10022 and WSCp10023. These primers include 25bp sequences homologous to those flanking the unique KpnI site in pTEV6. KpnI-linearized pTEV6 and each *divL* PCR product were combined in equimolar amounts and incubated at 50°C for 60 min in a reaction mixture containing T5 exonuclease, Phusion DNA polymerase, and Taq ligase (NEB) resulting plasmid pWSC10040 that incorporates Y550H into DivL(152-769).

***DivL(152-769) point mutant expression vectors*** DivL(152-769) point mutants were generated in pTEV5 using the Gibson DNA assembly method [3-5]. Each resulting plasmid encodes a hexahistidine tag and a TEV protease cleavage site (MSYYHHHHHHDYDIPTSENLYFQG) at the N-terminus of DivL(152-769) with a unique amino acid substitution as described below for Y550H.

**Y550H:** Codons 152-554 of *divL* were amplified by PCR using cosmid 2G1 as a template with primers WSCp10062 and WSCp62, and codons 546-769 were amplified as a separate PCR product using primers WSCp70 and WSCp10063. Codons 546-554 and the Y550H mutation were encoded within the 5' ends of primers WSCp62 and WSCp70 to provide sequence homology for seamless reconstitution of the intact DivL(152-769) ORF in the Gibson reaction. Primers WSCp10062 and WSCp10063 include 25bp sequences homologous to those flanking the unique NheI site in pTEV5. NheI-linearized pTEV5 and each *divL* PCR product were combined in equimolar amounts and incubated at 50°C for 60 min in a reaction mixture containing T5 exonuclease, Phusion DNA polymerase, and Taq ligase (NEB) resulting plasmid pWSC10040 that incorporates Y550H into DivL(152-769). DivL(152-769) point mutants R553A, T557N, Y562A, H579E, and A601L were generated in pTEV5 in a similar manner and details of their construction are described in Supplementary Table S4.

***pXYPC1-divL-yfp mutant expression vectors*** DivL-yfp point mutants were generated into the pXYFPC-1 vector that is designed to integrate at the xylose chromosomal locus using the Gibson DNA assembly method [3-5]. Each resulting plasmid encodes the full length *divL* coding sequence fused to yfp via a -SNVTRHRSAT- linker sequence with a unique amino acid substitution as summarized in Supplementary Table S5.

**Supplemental References**

1. Alley MR, Gomes SL, Alexander W, Shapiro L (1991) Genetic analysis of a temporally transcribed chemotaxis gene cluster in Caulobacter crescentus. Genetics 129: 333-341.

2. Ho SN, Hunt HD, Horton RM, Pullen JK, Pease LR (1989) Site-directed mutagenesis by overlap extension using the polymerase chain reaction. Gene 77: 51-59.

3. Gibson DG, Young L, Chuang RY, Venter JC, Hutchison CA, 3rd, et al. (2009) Enzymatic assembly of DNA molecules up to several hundred kilobases. Nat Methods 6: 343-345.

4. Hillson NJ, Rosengarten RD, Keasling JD (2012) j5 DNA assembly design automation software. ACS Synth Biol 1: 14-21.

5. Chen J, Densmore D, Ham TS, Keasling JD, Hillson NJ (2012) DeviceEditor visual biological CAD canvas. J Biol Eng 6: 1.
